# TGF-β1 suppresses the T-cell response in teleost fish by initiating Smad3- and Foxp3-mediated transcriptional networks

**DOI:** 10.1016/j.jbc.2022.102843

**Published:** 2022-12-26

**Authors:** Qian Zhang, Ming Geng, Kang Li, Haiyou Gao, Xinying Jiao, Kete Ai, Xiumei Wei, Jialong Yang

**Affiliations:** 1 State Key Laboratory of Estuarine and Coastal Research, School of Life Sciences, East China Normal University, Shanghai, China; 2 Laboratory for Marine Biology and Biotechnology, Qingdao National Laboratory for Marine Science and Technology, Qingdao, China

**Keywords:** adaptive immune, evolution, Foxp3, Smad3, T cell, teleost, transforming growth factor-β1, DMEM, Dulbecco’s modified Eagle’s medium, DPI, days post infection, FACS, flow cytometry and cell sorting, FBS, fetal bovine serum, FL, full-length, IL, interleukin, i.p., intraperitoneally, LPS, lipopolysaccharide, MP, mature peptide, PBS-T, PBS with 0.05% Tween-20, PHA, phytohaemagglutinin, qPCR, quantitative real-time RT-PCR, TGF-β1, transforming growth factor-β1

## Abstract

Transforming growth factor-β1 (TGF-β1) can suppress the activation, proliferation, and function of many T-cell subsets, protecting organisms from inflammatory and autoimmune disease caused by an overexuberant immune response. However, whether and how TGF-β1 regulates T-cell immunity in early vertebrates remain unknown. Here, using a Nile tilapia (*Oreochromis niloticus*) model, we investigated suppression of the T-cell response by TGF-β1 in teleost species. Tilapia encodes an evolutionarily conserved TGF-β1, the expression of which in lymphocytes is significantly induced during the immune response following *Edwardsiella piscicida* infection. Once activated, tilapia T cells increase TGF-β1 production, which in turn suppresses proinflammatory cytokine expression and inhibits T-cell activation. Notably, we found administration of TGF-β1 cripples the proliferation of tilapia T cells, reduces the potential capacity of Th1/2 differentiation, and impairs the cytotoxic function, rendering the fish more vulnerable to bacterial infection. Mechanistically, TGF-β1 initiates the TGF-βR/Smad signaling pathway and triggers the phosphorylation and nuclear translocation of Smad2/3. Smad3 subsequently interacts with several transcriptional partners to repress transcription of cytokines IL-2 and IFN-γ but promote transcription of immune checkpoint regulator CTLA4 and transcription factor Foxp3. Furthermore, TGF-β1/Smad signaling further utilizes Foxp3 to achieve the cascade regulation of these T-cell genes. Taken together, our findings reveal a detailed mechanism by which TGF-β1 suppresses the T cell–based immunity in Nile tilapia and support the notion that TGF-β1 had already been employed to inhibit the T-cell response early in vertebrate evolution, thus providing novel insights into the evolution of the adaptive immune system.

T cells play indispensable roles in tumor surveillance and infection elimination (1). However, dysregulation of these cells is highly associated with chronic infection and autoimmune diseases (2). Therefore, mammals such as mouse and human utilize sophisticated mechanisms to avoid an overexuberant T-cell response. For example, the inhibitory receptor PD-1 suppresses T-cell activation by compromising TCR signaling through dephosphorylating CD3ζ, ZAP-70, and PKCθ (3), whereas CTLA-4 inhibits T-cell response by competing the ligand binding with costimulation receptor CD28 (4). Meanwhile, the cell surface inhibitory molecule LAG3 (CD223) could either bind MHC class II to suppress the activation and proliferation of T cells (5) or bind Galectin-3 and LSECtin to affect T-cell function (6, 7). In addition, overexpression of V-domain Ig-containing inhibitor of T cell activation (VISTA) results in the defect of T-cell activation, proliferation, and cytokine production, but the blockade of VISTA has been found to enhance T-cell responses and exacerbate the development of experimental autoimmune encephalomyelitis *in vitro* (8, 9). Moreover, T_reg_ cells negatively control T-cell responses at least by secreting immunosuppressive cytokines interleukin (IL)-10 and transforming growth factor-β (TGF-β) (10, 11). Among which, IL-10 inhibits the expression of costimulatory molecules and MHC class II (12), suppresses the production of IFN-γ, IL-2, and TNF-α (13), compromises the differentiation and function of Th1, Th2, and Th17 cells (14), thus maintaining immune homeostasis and preventing excessive inflammatory responses.

TGF-β1 is another prototypical anti-inflammatory cytokine that plays central roles in preventing excessive inflammation and autoimmune disease. As an important member of the TGF-β superfamily, TGF-β1 can be produced by virtually all types of leukocytes (15, 16), and it exerts pleiotropic effects on proliferation, differentiation, adsorption, and programmed death of the immune cells, especially in many T-cell subsets such as CD8^+^ T cells, Th1, Th2, Th17, and T_reg_ cells (17, 18, 19, 20). Increasing advances in mammals have shown the potent inhibition of TGF-β1 on T-cell proliferation *via* multiple mechanisms, including suppressing IL-2 production (21), compromising c-Myc expression (22), and elevating cyclin-dependent kinase inhibitors (23). Mice with a T cell–specific deletion of the TGF-β1 developed lethal immunopathology in multiple organs, which is associated with the enhanced T-cell activation, proliferation, and Th1 and Th2 CD4^+^ T-cell differentiation (24). Meanwhile, TGF-β1 suppresses the development of Th17 cells–mediated encephalitis (25) and Th1 cells–mediated inflammatory-bowel disease in mice (26). In addition, TGF-β1 is also found to negatively control the innate immune system by inhibiting the activities of natural killer cells, macrophages, and neutrophils (27), indicating its pleiotropic regulation on immune cells and thus form a network for the negative immunomodulation (28).

The inhibitory function of TGF-β1 on T-cell immunity depends on the TGF-βR/Smad signaling. Upon binding the dimerized TGF-β1, TGF-βR2 of the receptor complex is autophosphorylated, which in turn phosphorylates the cytoplasmic juxta-membrane domain of TGF-βR1 (29). Phosphorylation of such domain converts it from a site that silences kinase activity to one that binds Smad proteins (30). The R-Smads (Smad2 and Smad3) are recruited and phosphorylated on two C-terminal Ser residues Ser-X-Ser (31) and then forms a trimer complex with the Co-Smad (Smad4). Subsequently, the Smad2-3-4 complex will translocate into the nucleus, where the R-Smad serves as an important transcription partner that cooperates with other transcription factors to promote target genes expression (32). In addition, Smad3 directly interacts with the promoter region of Foxp3 for its transcription, and Foxp3 further binds the fork-head DNA-binding elements of many T-cell required genes (*i.e.*, IL-2 and CD25) to suppress their expression (33). Considering the critical roles of TGF-β1 signaling in the anti-inflammatory response, a deficiency or mutation of either TGF-β1, its receptors, or signal transduction molecules will lead to spontaneous or excessive T-cell responses, which are highly associated with autoimmune diseases and immunopathology (34).

Although suppression of T-cell response by TGF-β1 has been widely elucidated in human and mouse, whether and how this cytokine negatively regulated T-cell immunity in early vertebrates remains unclear. Given that fish represent the lowest extant organisms to possess T cells, they are considered ideal models for studying T-cell evolution. In the past 2 decades, various T-cell subsets and their immunological functions in terms of cytotoxic, helper, and regulatory properties have been illustrated in many teleost species, such as zebrafish, rainbow trout, ginbuna crucian carp, and *et al.* (35, 36, 37). Mechanistically, using Nile tilapia *Oreochromis niloticus* as a model, we elucidated several signaling pathways (*i.e.*, Ca2^+^-NFAT, MAPK/ERK, NF-κB, and mTORC1) collectively underpin the activation, proliferation, and antibacterial immune function of teleost T cells (38, 39, 40, 41). However, whether the T-cell response in fish is tightly manipulated by some inhibitory strategy remains further investigation. Recently, TGF-β1 has been identified from many fish species such as zebrafish, carp, grass carp, crucian carp, goldfish, and tilapia (35, 37, 42, 43, 44, 45), and their expression in leukocytes can be induced by lipopolysaccharide (LPS), Poly I:C, Con A, or TNF-α (42, 44, 46, 47). Moreover, TGF-β1 significantly suppresses the LPS or TNFα-induced macrophage activation and IL-1β in goldfish, common carp, and grass carp (42, 44, 47), indicating their potential inhibition on innate immune response of teleost. In addition, the fact that the grass carp TGF-β1 is capable to compromise LPS or phytohaemagglutinin (PHA)-induced proliferation of peripheral blood lymphocyte (43) indicates fish TGF-β1 might also contribute to the negative regulation of adaptive immune response. Nevertheless, the detail modulation of TGF-β1 on teleost T-cell response and corresponding mechanism remain largely unknown. To this end, in present study, we showed that TGF-β1 negatively controlled the activation, proliferation, and function of T cells in tilapia, by initiating Smad3- and Foxp3-mediated transcriptional networks. Our findings imply that suppression of T-cell response by TGF-β1 is an evolutionarily ancient strategy, which appears before tetrapod emergence, and thus shed new insights into the evolution of T-cell immunity.

## Results

### An evolutionarily conserved TGF-β1 is present in Nile tilapia

**Figure 1:**
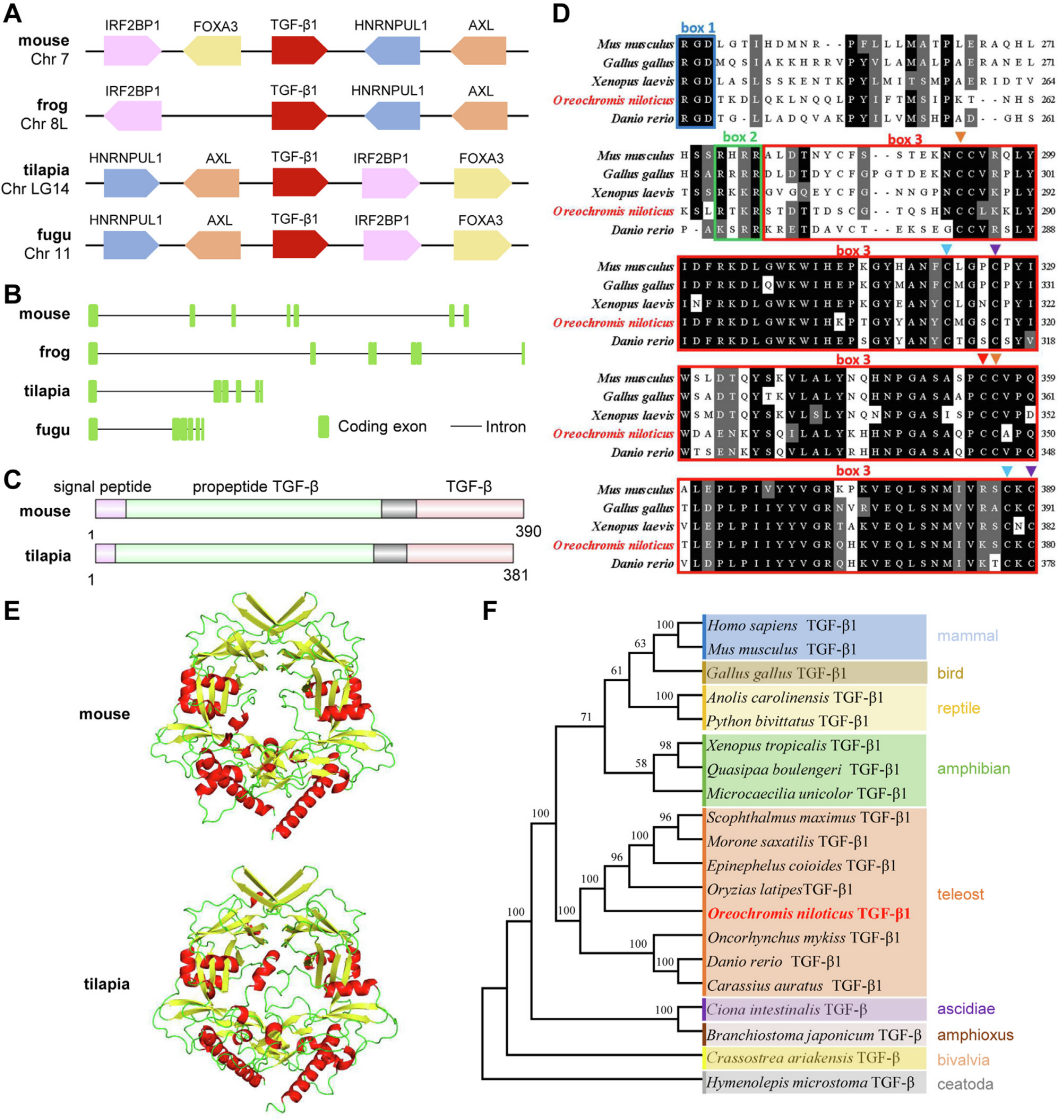
**The evolutionarily conserved TGF-β1 in Nile tilapia.***A*–*C*, comparison of TGF-β1 gene collinearity and chromosomal location (*A*), genomic structure (*B*), and domain organization (*C*) in indicated vertebrates. *D*, multisequence alignment analysis of TGF-β1 mature peptide domain from Nile tilapia and other animals. Amino acid residues with 80% identity are in *black*, and similar amino acids are in *gray*. Box1: integrin-binding site; box2: flynn protease recognition site; box3: TGF-β1 mature peptide domain. *Triangle*: cysteine residues that form disulfide bridges. *E*, the tertiary structures prediction of TGF-β1 from Nile tilapia and mouse were performed by SWISS-MODEL software. *F*, phylogenetic tree of TGF-β1 from the indicated species was constructed by the neighbor-joining algorithm in MEGAX software based on multiple sequence alignment by ClustalW. Bootstrap values of 1000 replicates (%) are indicated for the branches. The accession numbers of selected sequences are listed in Table S1. TGF-β1, transforming growth factor-β1.

Nile tilapia possesses a TGF-β1 gene on chromosome LG14. Analysis of comparative genomics revealed that both tilapia and fugu TGF-β1s locate in a chromosomal fragment composed of *HNRNPUL1-AXL-TGF-β1-IRF2BP1-FOXA3* genes (Fig. 1*A*). Despite undergoing some rearrangement, the adjacent genes are identical in mouse; and the same is almost true in frog, with only an exception that the FOXA3 is missing (Fig. 1*A*). Tilapia and fugu TGF-β1s contain six coding-exons and five introns, whereas in frog and mouse, it is seven coding-exons and six introns (Fig. 1*B*). The tilapia TGF-β1 contains 381 amino acids, encoding a signaling peptide, a pro-peptide TGF-β1 domain, and a TGF-β1 domain; and the organization of these domains is similar to that in mouse homolog (Fig. 1*C*). The primary structure of tilapia TGF-β1, especially that in the TGF-β1 mature peptide (MP) domain is evolutionarily conserved (Fig. 1*D*). In addition, these vertebrates’ TGF-β1s possess conserved functional motifs, including the integrin-binding site, furin-like peptidase, and seven cysteine residues that form three disulfide bridges and one interchain disulfide bridge (Fig. 1*D*). More importantly, tilapia and mouse TGF-β1s share similar tertiary structures (Fig. 1*E*). In a neighbor-joining phylogenetic tree, the mammalian TGF-β1 first clustered into a clade with the homologs in amphibians, reptiles and then formed a sister group with the clade formed by teleost TGF-β1 (Fig. 1*F*). The clustering of tilapia TGF-β1 with its homologs in other teleost indicated their close evolutionary relationship (Fig. 1*F*). Taken together, these results suggested that tilapia TGF-β1 is an evolutionarily conserved cytokine, which may have immunosuppressive functions similar to that in mammals.

### TGF-β1 takes part in the lymphocyte-mediated adaptive immune response of tilapia

**Figure 2:**
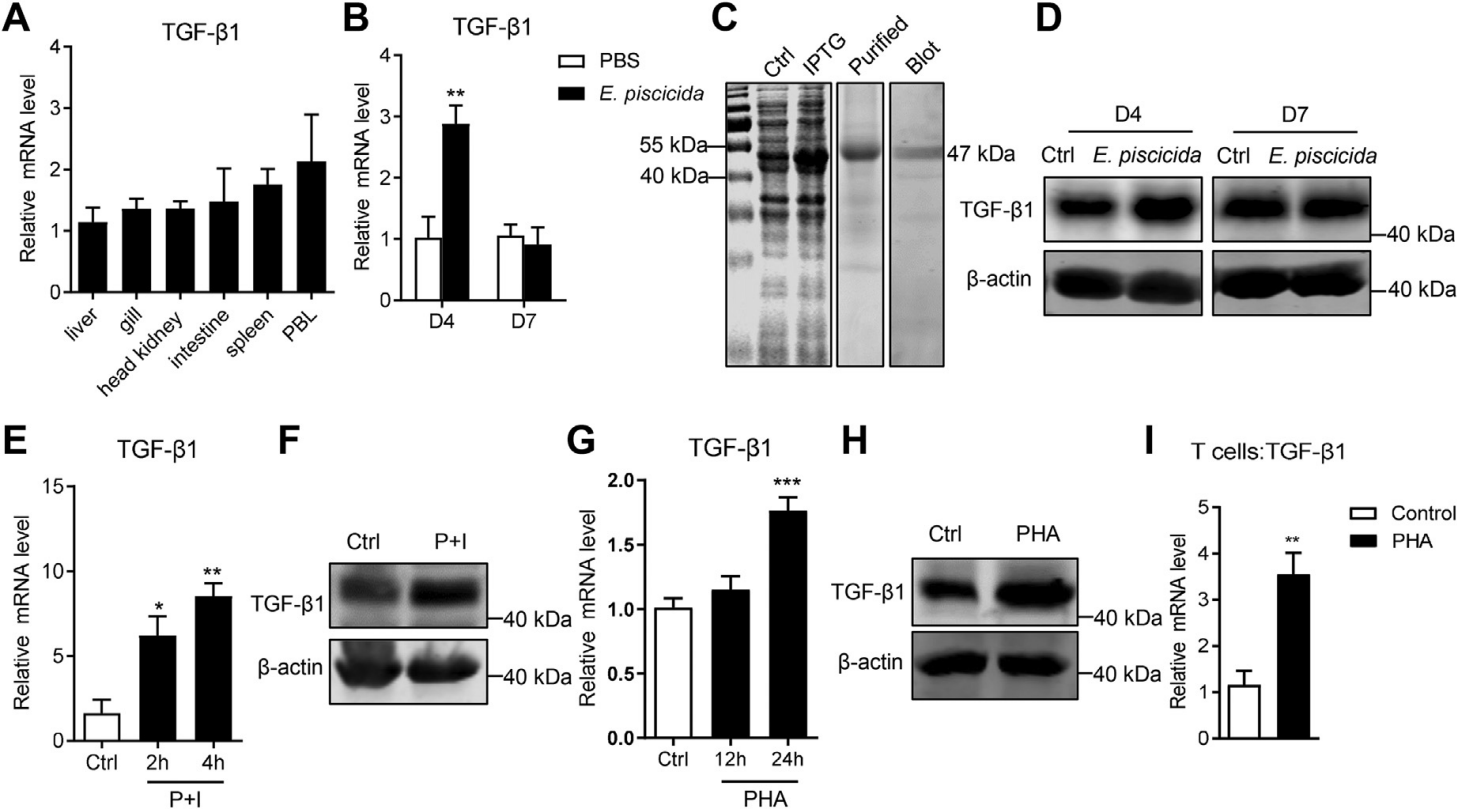
**TGF-β1 participates in the adaptive immune response of tilapia.***A*, TGF-β1 expression in the indicated tissues was analyzed by qPCR, n = 5. *B*, Nile tilapia was *i.p.* injected with *E. piscicida* or PBS, and spleen lymphocytes were harvested on the indicated days after infection. Relative mRNA levels of TGF-β1 was determined by qPCR, n = 6. *C*, recombinant and purification of Nile tilapia FL-TGF-β1 in the *Escherichia coli* prokaryotic recombinant system were analyzed by SDS-PAGE, and the interaction between TGF-β1 protein in spleen lymphocytes and anti-FL-TGF-β1 polyclonal antibody was determined by Western blotting. *D*, the protein level of TGF-β1 in spleen lymphocytes at different timepoints after *E. piscicida* infection was examined by Western blotting. *E*–*H*, the spleen lymphocytes were stimulated by P + I (*E* and *F*) or PHA (*G* and *H*) *in vitro*. The expression level of TGF-β1 was determined by qPCR at indicated times (*E* and *G*) and by Western blotting at 4 h (*F*) or 12 h (*H*). *I*, sorted spleen CD3^+^ T cells were stimulated with PHA for 12 h, and TGF-β1 expression was determined by qPCR, n = 4 to 5. The above experiments were repeated at least two independent times. ∗*p* < 0.05, ∗∗*p* < 0.01, ∗∗∗*p* < 0.001, determined by a two-tailed Student’s *t* test. FL, full-length; i.p., intraperitoneally; qPCR, quantitative real-time RT-PCR; TGF-β1, transforming growth factor-β1.

TGF-β1 was constitutively expressed in the liver, gill, head kidney, intestine, spleen, and peripheral blood of tilapia, with similar expression levels (Fig. 2*A*). To determine whether TGF-β1 takes part in lymphocyte-mediated adaptive immunity of tilapia, we infected the fish with pathogenic bacteria *E. piscicida*. The transcription level of TGF-β1 in spleen lymphocytes was significantly upregulated at 4-days post infection (DPI) and returned to the original level at 7 DPI (Fig. 2*B*). In order to examine the TGF-β1 expression at protein level, we prepared the recombinant protein of full-length (FL) TGF-β1 and immunized mice to obtain anti-TGF-β1 polyclonal antibody, which could specifically recognize TGF-β1 in tilapia lymphocytes (Fig. 2*C*). Consist with the mRNA level, TGF-β1 protein was markedly induced at 4-days after infection and then dropped back to normal level at 7 DPI (Fig. 2*D*). Then, we activated the spleen lymphocytes *in vitro* with phorbol 12-myristate 13-acetate plus ionomycin (P + I) and found that both the mRNA and protein levels of TGF-β1 were obviously upregulated compared with the control counterparts (Fig. 2, *E* and *F*), confirming the production of TGF-β1 by the lymphocytes in tilapia. To further explore the participation of TGF-β1 in T-cell response, we stimulated spleen lymphocytes with the T-cell–specific mitogen PHA. Upon PHA-induced T-cell activation, both mRNA and protein levels of TGF-β1 were concordantly elevated (Fig. 2, *G* and *H*). Moreover, the mRNA expression of TGF-β1 in sorted CD3^+^ T cells was significantly upregulated (Fig. 2*I*), indicating the cellular source of TGF-β1 by the T-cell lineage in tilapia. Overall, these findings suggest that TGF-β1 participates in the lymphocytes- or T cells–mediated adaptive immune response of Nile tilapia.

### TGF-β1 restrains proinflammatory cytokines and T-cell activation of tilapia

**Figure 3:**
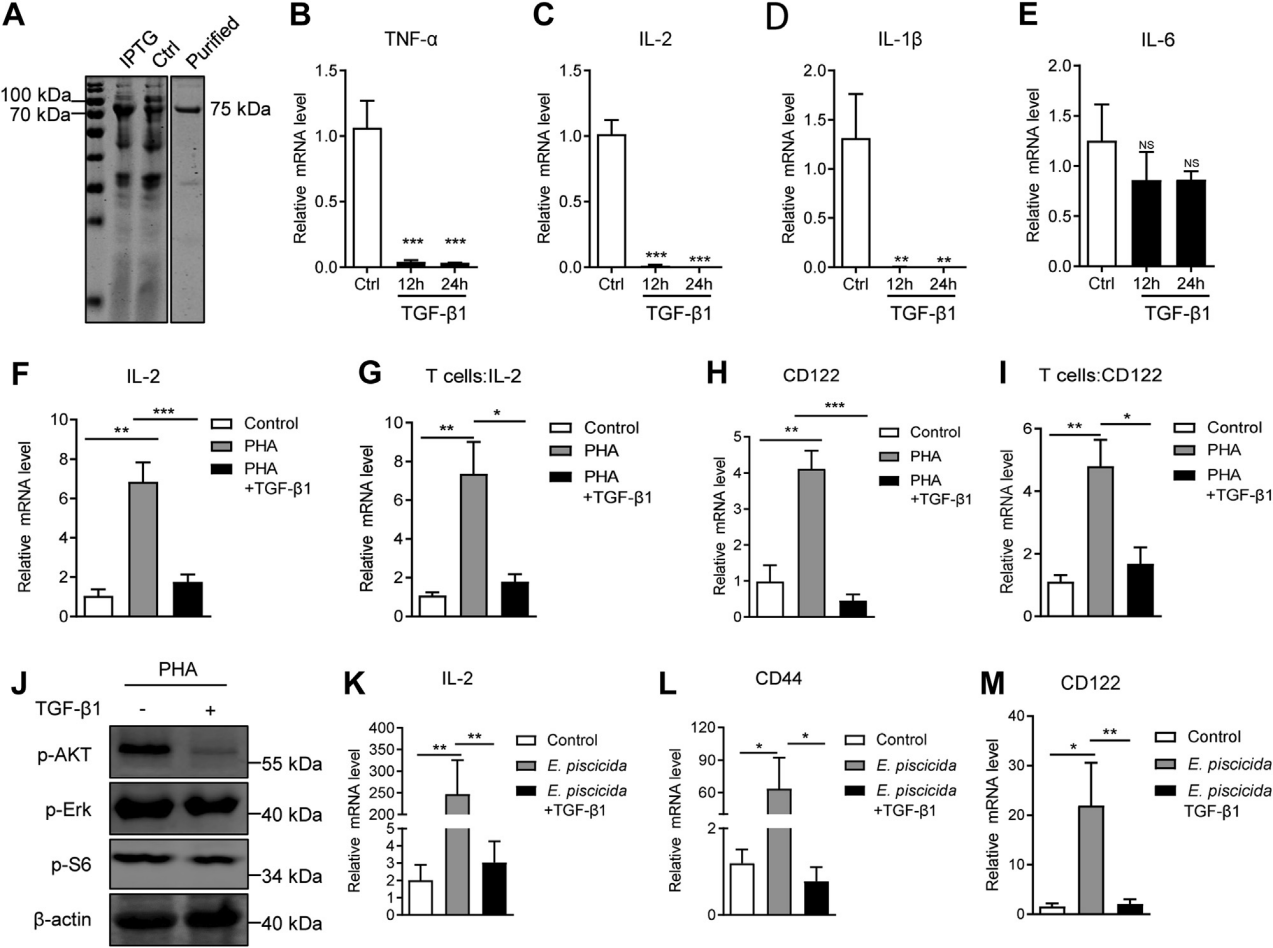
**Tilapia TGF-β1 inhibits proinflammatory cytokines expression and T-cell activation.***A*, SDS-PAGE analysis shows the recombinant and purification of recombinant Nile tilapia MP-TGF-β1 in *Escherichia coli*. *B*–*E*, the expression levels of TNF-α (*B*), IL-1β (*C*), IL-2 (*D*), and IL-6 (*E*) in spleen lymphocytes upon MP-TGF-β1 stimulation *in vitro* at indicated timepoints, n = 6. *F*–*J*, the spleen lymphocytes (*F*, *H* and *J*) or sorted CD3^+^ T cells (*G* and *I*) were simulated by PHA with or without recombinant MP-TGF-β1 for 12 h. The expression levels of IL-2 and CD122 were examined by qPCR, n = 4 to 6. And phosphorylation levels of indicated protein were examined by Western blotting (*J*). *K*–*M*, Nile tilapia that infected by *E. piscicida* was *i.p.* injected with MP-TGF-β1. The expression levels of IL-2 (*K*), CD44 (*L*), and CD122 (*M*) in spleen lymphocytes were examined by qPCR on 5-days after infection, n = 6. The above experiments were repeated at least two independent times. ∗*p* < 0.05, ∗∗*p* < 0.01, ∗∗∗*p* < 0.001, determined by a two-tailed Student’s *t* test. IL, interleukin; i.p., intraperitoneally; MP, mature peptide; qPCR, quantitative real-time RT-PCR; TGF-β1, transforming growth factor-β1.

We next sought to know the exact function TGF-β1 exerted in the cellular immunity. Firstly, the recombinant mature TGF-β1 protein was prepared (Fig. 3*A*) to treat spleen lymphocytes *in vitro*. TGF-β1 treatment markedly impaired the expression of the proinflammatory cytokines TNF-α, IL-2, and IL-1β (Fig. 3, *B*–*D*), but not IL-6 (Fig. 3*E*), indicating its potential suppression on the inflammation of tilapia. Considering IL-2 is indispensable for the proper activation, proliferation, and survival of T cells, we then examined the regulation of TGF-β1 on the T-cell response. PHA stimulation induced a robust T-cell activation, evidencing by the upregulated expression of IL-2 (Fig. 3, *F* and *G*), and the IL-2 receptor CD122 (Fig. 3, *H* and *I*), in both spleen lymphocytes and sorted CD3^+^ T cells. However, administration of extra TGF-β1 markedly impaired the PHA-induced elevation of these T-cell activation markers (Fig. 3, *F*–*I*) and reduced the phosphorylation of AKT, S6, and ERK1/2 (Fig. 3*J*), which are of paramount importance for T-cell activation in tilapia (41). Consist with these *in vitro* evidences, *in vivo* administration of tilapia with recombinant TGF-β1 during *E. piscicida* infection severely impaired the inducible expression of IL-2 (Fig. 3*K*) and CD44 and CD122 (Fig. 3, *L* and *M*). Altogether, our findings suggest that TGF-β1 negatively regulates the expression of proinflammatory cytokines and T-cell activation in tilapia.

### TGF-β1 inhibits proliferation and survival of tilapia T cells

**Figure 4:**
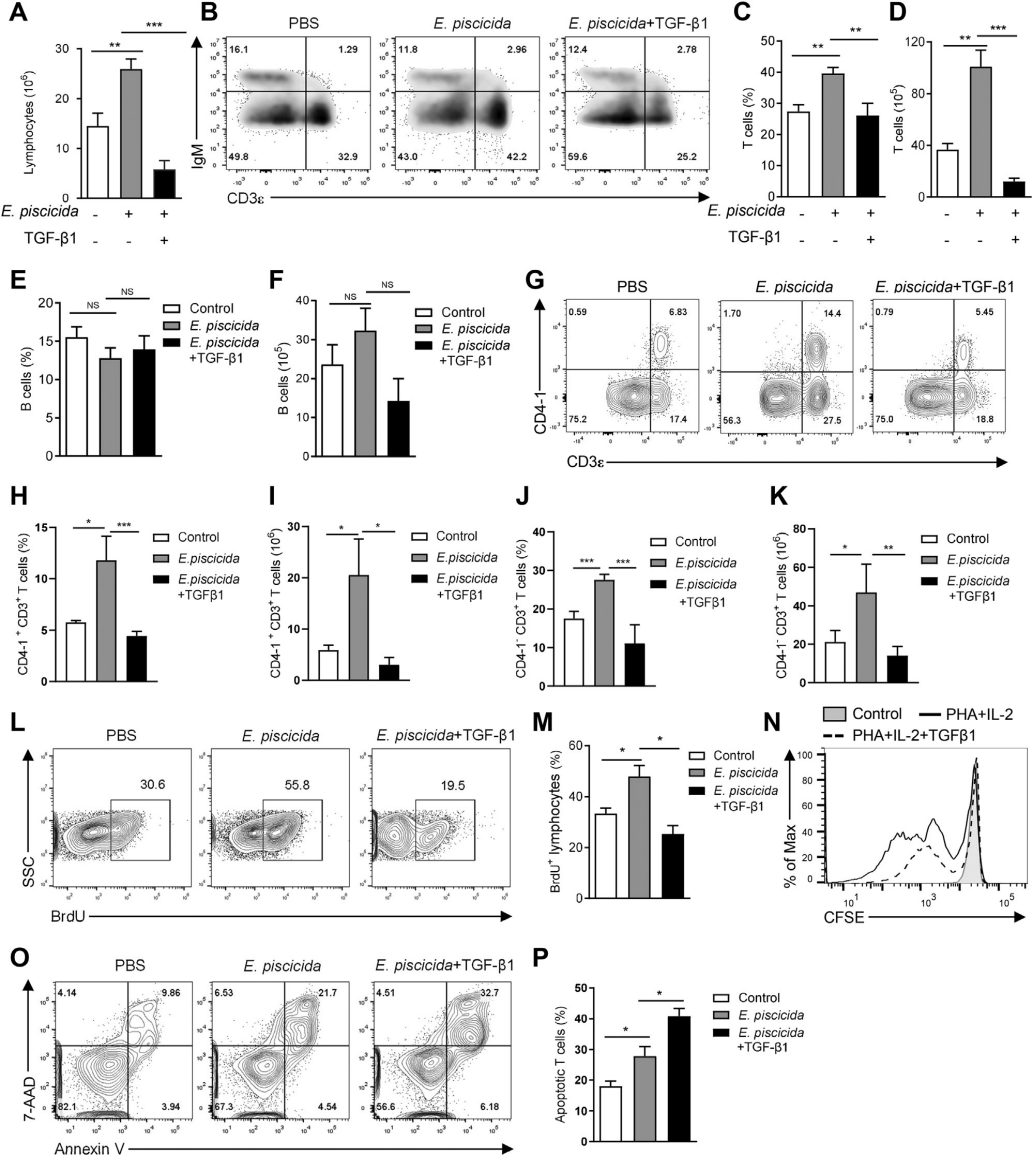
**TGF-β1 compromises T-cell proliferation and survival in tilapia.** The tilapia individuals infected with *E. piscicida* were *i.p.* injected with or without recombinant MP-TGF-β1, and spleen lymphocytes were harvested for assay. *A*, the numbers of spleen lymphocytes on 7 DPI, n = 6. *B*–*F*, spleen lymphocytes were stained by IgM and CD3 antibody and analyzed by flow cytometry on 7 DPI. Representative density plots (*B*), percentage (*C* and *E*), and absolute number (*D* and *F*) of CD3^+^ T cells and IgM^+^ B cells were shown, n = 6. *G*–*K*, spleen lymphocytes were stained by CD4-1 and CD3 antibodies and analyzed by flow cytometry on 7 DPI. Representative FACS plots (*G*), percentage (*H* and *J*), and absolute number (*I* and *K*) of CD4-1^+^CD3^+^ T cells, and CD4-1^−^CD3^+^ T cells were shown, n = 5 to 6. *L* and *M*, the tilapia individuals *i.p.* injected with BrdU 1 day before were sacrificed on 5 DPI. Representative contour plots (*L*) and percentage (*M*) of BrdU^+^ lymphocytes in spleen, n = 3. *N*, CFSE-labeled lymphocytes isolated from spleen of healthy tilapia were stimulated with PHA and recombinant IL-2 with or without MP-TGF-β1 for 72 h, and overlaid histogram shows the proliferation of the CD3^+^ T cells. *O* and *P*, representative contour plot of 7AAD and Annexin V staining in gated spleen T cells (*O*) and percentage of apoptotic T cells (*P*, n = 5) on 7 DPI. The above experiments were repeated at least two independent times. ∗*p* < 0.05, ∗∗*p* < 0.01, ∗∗∗*p* < 0.001, determined by a two-tailed Student’s *t* test. DPI, days post infection; FACS, flow cytometry and cell sorting; IL, interleukin; i.p., intraperitoneally; MP, mature peptide; TGF-β1, transforming growth factor-β1.

Considering TGF-β1 suppresses the T-cell activation of tilapia, we further sought to know its potential effects on the expansion of this cell lineage. We administrated the tilapia with recombinant TGF-β1 during *E. piscicida* infection. At 5 DPI, spleen lymphocytes in the bacterial infected fish were obviously increased than those in control group, whereas such lymphocytes expansion was dramatically impaired in the presence of extra TGF-β1 (Fig. 4*A*). Within the lymphocyte population, bacterial infection significantly increased the expansion of CD3^+^ T cells (Fig. 4, *B*–*D*), while treatment with TGF-β1 markedly reduced both T-cell percentage and number (Fig. 4, *B*–*D*). In contrast, IgM^+^ B cells did not fluctuate significantly upon bacterial infection or TGF-β1 administration at 5 DPI (Fig. 4, *B*, *E*, and *F*). Furthermore, additional TGF-β1 also compromised the *E. piscicida*–induced expansion of CD4-1^+^CD3^+^ T cells and CD4-1^−^CD3^+^ T cells (Fig. 4, *G*–*K*). To elucidate how TGF-β1 suppressed the expansion of tilapia T cells, we respectively examined the proliferation and apoptosis of these cells. *E. piscicida* infection increased the BrdU incorporation in spleen lymphocytes, whereas TGF-β1 treatment dramatically reduced the percentage of BrdU^+^ cells (Fig. 4, *L* and *M*), indicating the inhibition of lymphocytes proliferation by TGF-β1. This was further confirmed by the *in vitro* CFSE dilution assay, in which a combinated stimulation of spleen lymphocytes with PHA and recombinant IL-2 induced a robust T-cell proliferation, which was markedly inhibited by the addition of TGF-β1 (Fig. 4*N*). Meanwhile, pathogenic infection induced an obvious apoptosis of tilapia T cells, while extra TGF-β1 further enhanced such kind of programmed T-cells death (Fig. 4, *O* and *P*). Therefore, these results demonstrate that TGF-β1 suppresses the expansion of tilapia T cells *via* compromising proliferation and survival.

### TGF-β1 suppresses the effector function of tilapia T cells

**Figure 5:**
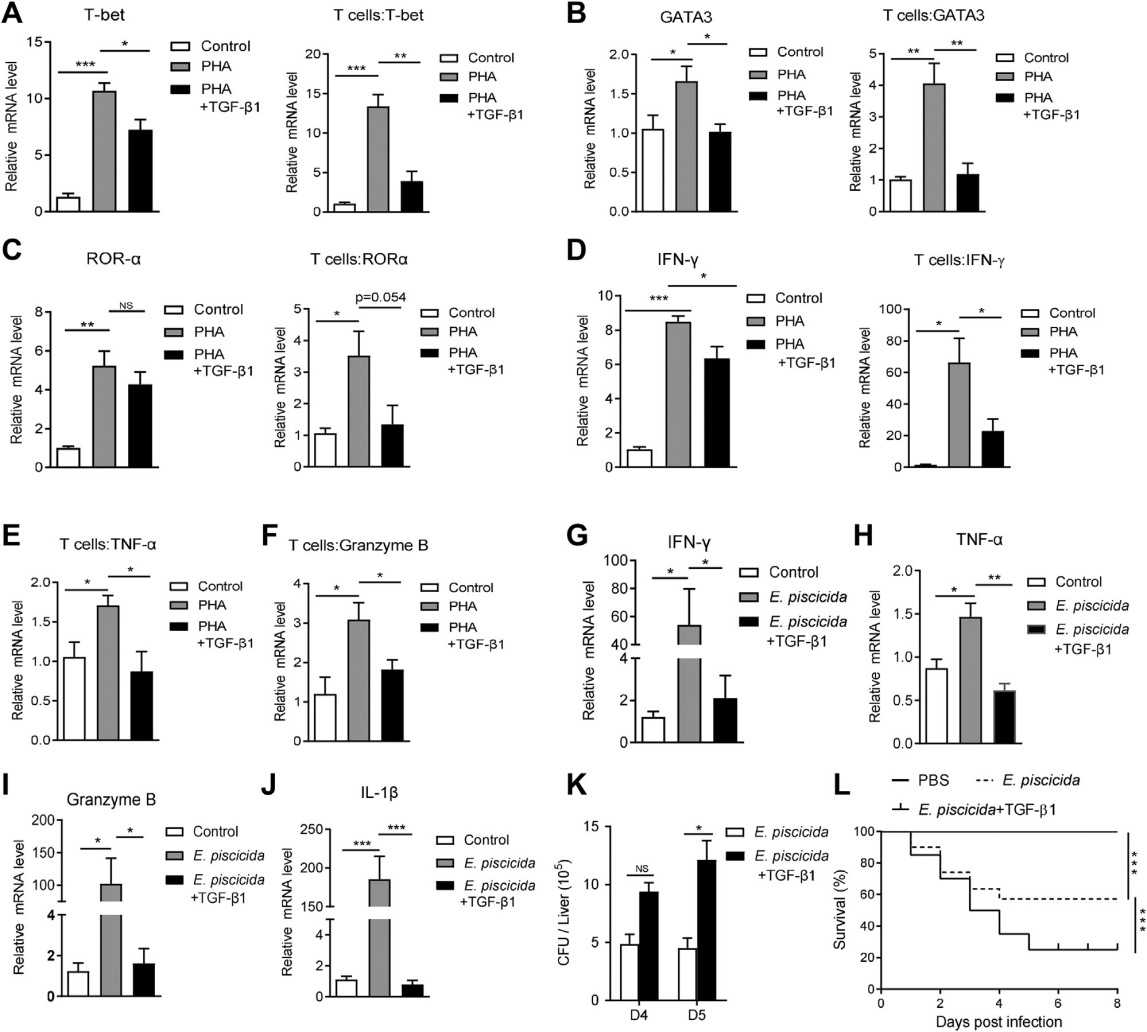
**TGF-β1 compromises the T-cell function of tilapia.***A*–*F*, the spleen lymphocytes or sorted CD3^+^ T cells were simulated by PHA with or without recombinant MP-TGF-β1. The relative mRNA levels of indicated molecules were examined by qPCR at 12 h, n = 6. *G*–*J*, the tilapia individuals infected with *E. piscicida* were *i.p.* injected with or without recombinant MP-TGF-β1. Relative mRNA levels of indicated molecules in spleen lymphocytes on 7 DPI were examined by qPCR, n = 6. *K*, *E.piscicida* titers in the liver on 4 and 5 DPI, n = 4. *L*, Kaplan-Meier survival plot indicated the survival percentage of Nile tilapia postinfection, n = 20. The above experiments were repeated at least two independent times. ∗*p* < 0.05, ∗∗*p* < 0.01, ∗∗∗*p* < 0.001, determined by a two-tailed Student’s *t* test. DPI, days post infection; i.p., intraperitoneally; MP, mature peptide; qPCR, quantitative real-time RT-PCR; TGF-β1, transforming growth factor-β1.

T cells differentiate into various subsets to perform distinct effector functions according to the antigen signals and microenvironmental cues (48). Here, we investigated the effects of TGF-β1 on the differentiation capacity of tilapia T cells. Upon PHA stimulation, the mRNA expression levels of T-bet, GATA3, and RORα were obviously upregulated in spleen lymphocytes or sorted CD3^+^ T cells (Fig. 5, *A*–*C*), indicating the potential ability of tilapia T cells to differentiate into Th1, Th2, and Th17 subsets. Once administrated with TGF-β1, their inducible expression was markedly impaired (Fig. 5, *A*–*C*), suggesting that TGF-β1 suppresses the potential differentiation of T helper cells in tilapia. In spleen lymphocytes or CD3^+^ T cells, TGF-β1 dampened the PHA-induced expression of the proinflammatory cytokines IFN-γ and TNF-α (Fig. 5, *D* and *E*) and the cytotoxic gene Granzyme B (Fig. 5*F*). Similar with these *in vitro* evidences, *E. piscicida* infection also upregulated the expression of these effector genes, and such induction was obviously impaired once tilapia were treated with recombinant TGF-β1 (Fig. 5, *G*–*J*). Since the inducible expression of TNF-α, IFN-γ, and Granzyme B are highly associated to T-cell cytotoxicity (17), our results implied that TGF-β1 might inhibit the effector function of tilapia T cells to eliminate pathogenic infection. The impaired T-cell differentiation and cytotoxic function collectively made the tilapia disable to control the invading bacteria (Fig. 5*K*) and caused a high mortality during the *E. piscicida* infection (Fig. 5*L*). Overall, these findings suggest that tilapia TGF-β1 is an anti-inflammatory cytokine suppressing the effector function of T cells.

### Tilapia utilizes conserved TGF-β1R/Smad pathway to sense TGF-β1 signaling

**Figure 6:**
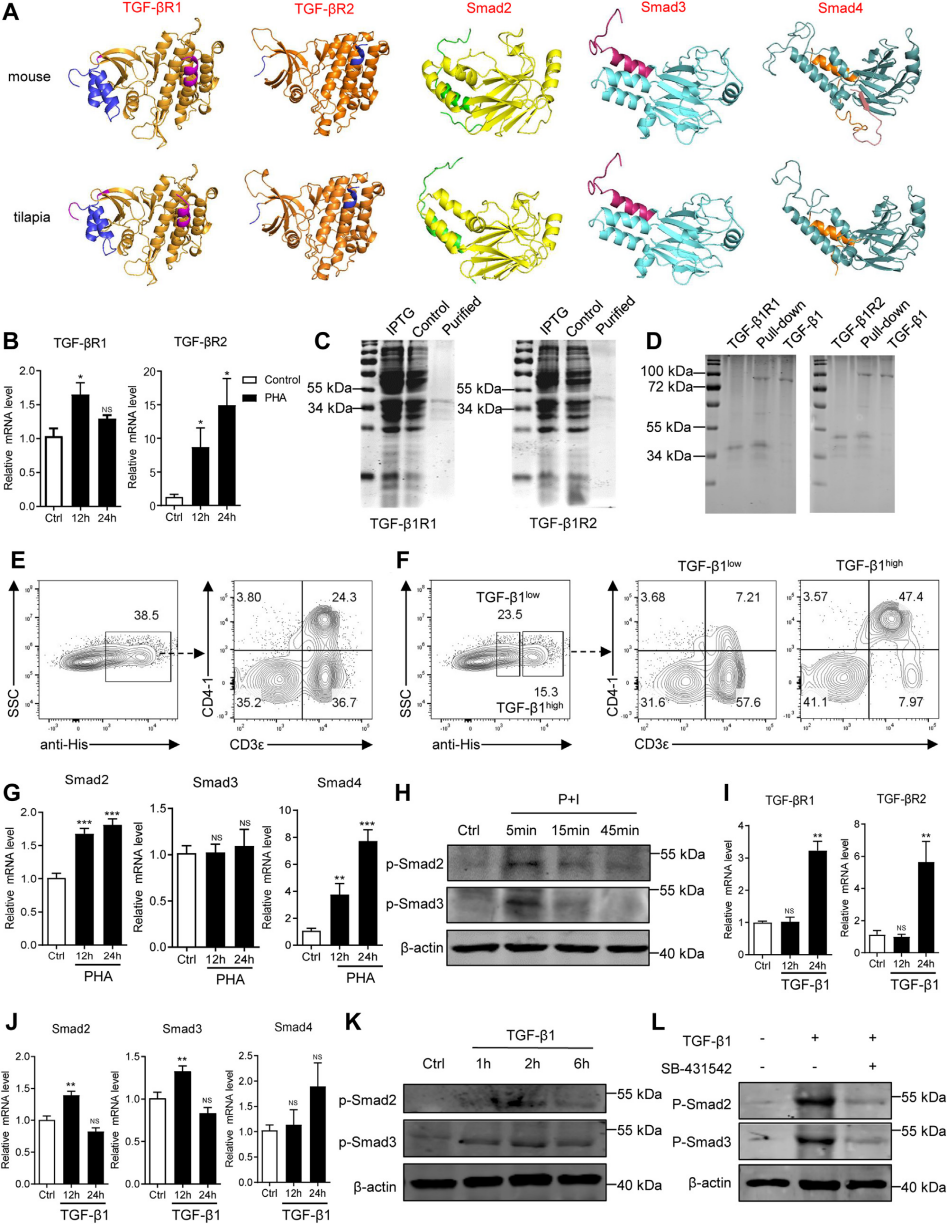
**Tilapia employs TGF-β1R/Smad axis to sense TGF-β1 signaling.***A*, comparison of the predicted tertiary structures of TGF-β1R1, TGF-β1R2, Smad2, Smad3, and Smad4 from Nile tilapia and mouse. The accession numbers of selected sequences are listed in Table S1. *B*, spleen lymphocytes were stimulated with PHA, and mRNA expression of TGF-βR1 and TGF-βR2 were examined by qPCR at indicated time. *C*, the recombinant GST-tag TGF-βR1 or TGF-βR2 was expressed by *Escherichia coli* prokaryotic recombinant system. *D*, interaction between the recombinant His-tag TGF-β1 and GST-tag TGF-βR1 or TGF-βR2 was examined by GST-pull down assay and shown by SDS-PAGE. *E*, spleen lymphocytes were incubated with recombinant His-tag-TGF-β1 and then stained with the anti-His, anti-tilapia CD4-1, and anti-tilapia CD3 mAbs. Flow cytometry showing the frequency of CD4-1^+^CD3^+^ T cells or CD4-1^−^CD3^+^ T cells among TGF-β1–binding lymphocytes. *F*, the same flow cytometry plot of (*E*) *left panel* was reused to analyze the frequency of CD4-1^+^CD3^+^ T cells or CD4-1^−^CD3^+^ T cells among TGF-β1^high^ and TGF-β1^low^ populations. *G*–*K*, spleen lymphocytes were stimulated with PHA (*G*), P + I (*H*), or recombinant MP-TGF-β1 (*I*–*K*) for indicated times. Relative mRNA (*G*, *I* and *J*, n = 6) or phosphorylation (*H* and *K*) levels of indicated molecules were determined by qPCR or Western blotting. *L*, spleen lymphocytes were stimulated with recombinant MP-TGF-β1 with or without TGF-β1 inhibitor for 2 h, and Western blotting assay shows the phosphorylation levels of indicated molecules. The above experiments were repeated at least two independent times. ∗*p* < 0.05, ∗∗*p* < 0.01, ∗∗∗*p* < 0.001, determined by a two-tailed Student’s *t* test. MP, mature peptide; qPCR, quantitative real-time RT-PCR; TGF-β1, transforming growth factor-β1.

Next, we sought to know how TGF-β1 exerts the immunosuppressive functions in tilapia. Mammalian TGF-β1 triggers intracellular signal transduction by binding to the TGF-β receptor complex consisting of TGF-βR1 and TGF-βR2 (29, 49) and then activates downstream Smad pathway (50). An in-depth search in the genome database confirmed the presence of TGF-βR1, TGF-βR2, Smad2, Smad3, and Smad4 in Nile tilapia. Like the mouse homologs, tilapia TGF-βR1 consists of an Activin domain and a GS domain in the extracellular fragment and serine/threonine kinase catalytic (S/TKc) domain in the intracellular part; while TGF-βR2 contains an extracellular TGFβR2 domain and an intracellular S/TKc domain (Fig. S1*A*). The evolutionarily conserved domain organization was also found in Smad molecules, because tilapia Smad2, Smad3, and Smad4, and also their counterparts in mouse, all encode MH1 and MH2 domains (Fig. S1*B*). The amino acid sequences of tilapia TGF-βR1, TGF-βR2, Smad2, Smad3, and Smad4, especially those encoding the functional domains, are conserved across the vertebrates (Figs. S2 and S3). Moreover, all these tilapia components adopt tertiary structures similar to that in mouse (Fig. 6*A*), and they are closely clustered with their respective counterparts from other teleosts in the phylogenetic trees (Fig. S4). These results thus suggest an intact and evolutionarily conserved TGF-βR/Smad pathway existing in Nile tilapia.

Similar to TGF-β1, these downstream components were widely expressed in the lymphoid-associated tissues/organs, including liver, gill, head kidney, intestine, spleen, and peripheral blood (Fig. S5). Upon PHA-induced T-cell activation, mRNA expressions of TGF-βR1 and TGF-βR2 were markedly upregulated in spleen lymphocytes (Fig. 6*B*). Then, the recombinant GST-tag TGF-βR1 and TGF-βR2 proteins were prepared to confirm their interaction with TGF-β1 (Fig. 6*C*). The GST pull-down assay showed that both TGF-βR1 and TGF-βR2 could interact with TGF-β1 *in vitro* (Fig. 6*D*). To further confirm the binding of TGF-β1 on tilapia T cells, spleen lymphocytes were incubated with the recombinant His-tag TGF-β1 and then stained with anti-His, anti-CD4-1, and anti-CD3 mAbs. About 38% of the lymphocytes could bind TGF-β1; while within the TGF-β1–binding lymphocytes, about 24% were CD4-1^+^CD3^+^ T cells, and about 36% were CD4-1^−^CD3^+^ T cells (Fig. 6*E*). In addition, we found that most CD4-1^+^CD3^+^ TGF-β1–binding T cells distributed in TGF-β1^high^ population, but CD4-1^−^CD3^+^ TGF-β1–binding T cells were mainly from TGF-β1^low^ gate (Fig. 6*F*), suggesting CD4-1^+^CD3^+^ T cells exhibited stronger TGF-β1–binding ability than CD4-1^−^CD3^+^ T cells. Downstream of TGF-β signaling, transcription levels of Smad2 and Smad4, but not Smad3, were induced by PHA stimulation (Fig. 6*G*). Meanwhile, the phosphorylation of Smad2 and Smad3 was remarkably enhanced after P + I stimulation (Fig. 6*H*), thus indicating the robust activation of TGF-βR/Smad pathway during T-cell activation. Furthermore, treatment of spleen lymphocytes with recombinant TGF-β1 elevated the transcription levels of TGF-βR1, TGF-βR2, Smad2, and Smad3 (Fig. 6, *I* and *J*) and also the phosphorylation of Smad2 and Smad3 (Fig. 6*K*). In contrast, once TGF-βR2 was blocked by specific inhibitor, TGF-β1–induced phosphorylation of Smad2 and Smad3 was significantly impaired (Fig. 6*L*), demonstrating the indispensable role of TGF-β1 receptor in initiating the Smad signaling. Altogether, these results suggest that Nile tilapia utilizes an evolutionarily conserved TGF-βR/Smad pathway to sense TGF-β1 signaling.

### Smad3 transcriptionally controls T-cell immunity of tilapia

**Figure 7:**
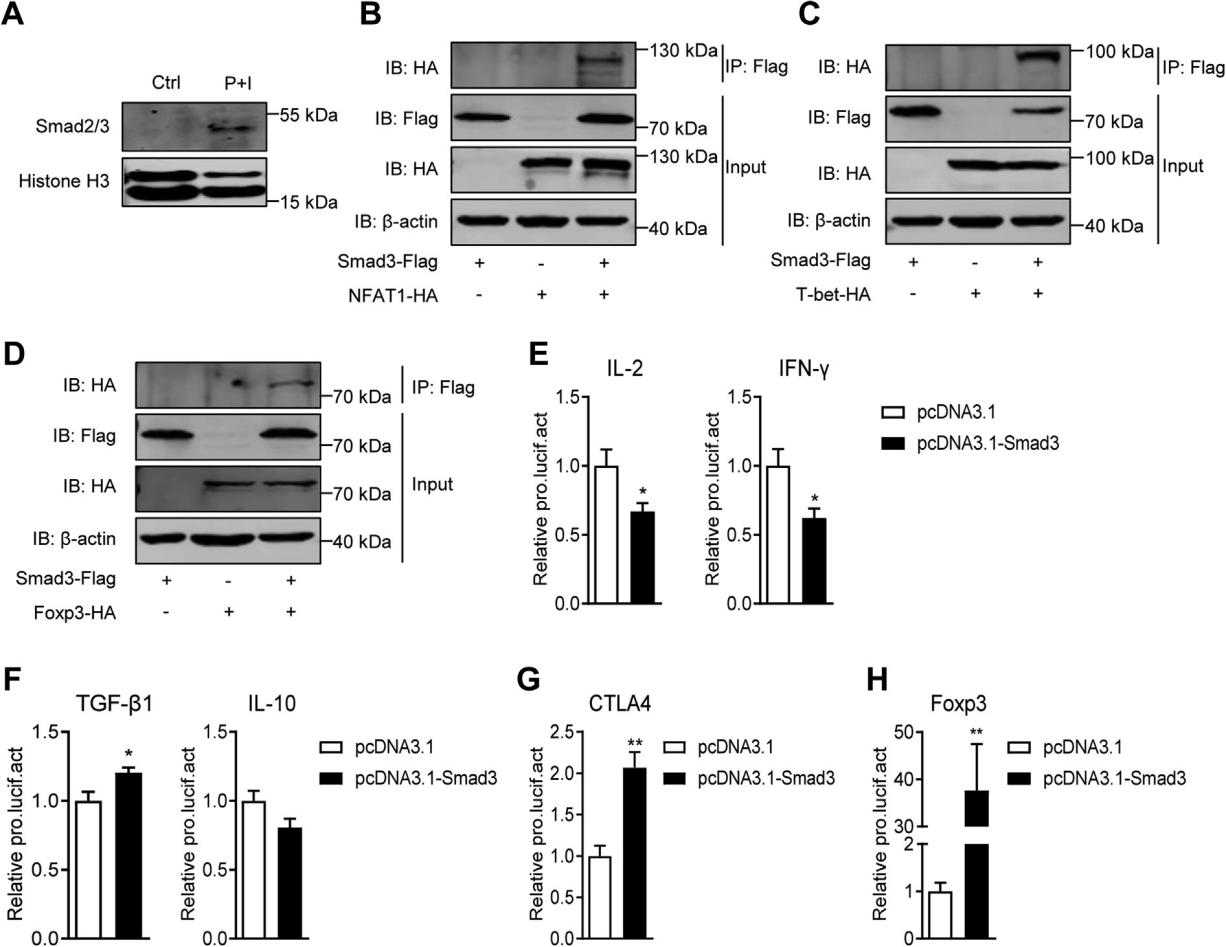
**Smad3 regulates the transcription of T-cell genes in tilapia.***A*, Western blotting assay shows the protein levels of Smad2/3 and Histone H3 in the nucleus of spleen lymphocytes that stimulated with P + I or not for 20 min. *B*–*D*, HEK 293T cells cotransfected with Smad3-Flag-pEGFP-C1 plasmid and indicated partner-HA-pEGFP-C1 were subjected to Western blotting directly for input control or after immunoprecipitation with anti-Flag Ab-conjugated agarose beads. The interaction of Smad3 with NFAT1 (*B*), T-bet (*C*), and Foxp3 (*D*) were examined. *E*–*H*, HEK 293T cells were cotransfected with tilapia Smad3 and pGL3-IL-2 (IFN-γ, TGF-β1, IL-10, CTLA4, or Foxp3) promoter. The LUC activities were assessed at 48 h post transfection, n = 4. The above experiments were repeated at least two independent times. ∗*p* < 0.05, ∗∗*p* < 0.01, determined by a two-tailed Student’s *t* test. IL, interleukin; TGF-β1, transforming growth factor-β1.

Upon activation, Smad complex will translocate into the nucleus, where it associates with other transcription factors, coactivators, or corepressors to regulate target gene expression (33). Therefore, we explored the transcriptional role of Smad in tilapia. Once tilapia spleen lymphocytes were activated by P + I stimulation, protein level of Smad2/3 was obviously enhanced in the nucleus, suggesting the occurrence of R-Smad nuclear translocation (Fig. 7*A*). Using co-immunoprecipitation (Co-IP) approach, we revealed that tilapia Smad3 can not only interact with coactivator NFAT1 and T-bet (Fig. 7, *B* and *C*) but also associate with corepressor Foxp3 (Fig. 7*D*). Furthermore, the LUC activity driven by the promoter of IL-2 or IFN-γ was significantly reduced by overexpression of tilapia Smad3 (Fig. 7*E*). Instead, the promoter activities of anti-inflammatory cytokines TGF-β1 and IL-10 was slightly enhanced or not changed (Fig. 7*F*). Notably, transcriptional activities of the inhibitory receptor CTLA4 and transcription factor Foxp3, which are important for T_reg_ function, were dramatically increased once Smad3 was overexpressed (Fig. 7, *G* and *H*). Overall, these results suggest that Smad3 cooperates with its partners to regulate the transcription of T-cell immunological genes in tilapia.

### TGF-β1 signaling employs Foxp3 to transcriptionally restrain tilapia T-cell response

Another acknowledged approach by which mammalian TGF-β1 suppresses T-cell response is to induce Foxp3 expression (51, 52). However, whether TGF-β1 restrains fish T-cell immunity by inducing Foxp3 expression remains unclear. The cDNA of Nile tilapia Foxp3 has been cloned in previous study (53). Here, we revealed that Foxp3 gene located on chromosome LG20 of tilapia, and it is consistently downstream of GPR173 in frog, tilapia, and puffer fish (Fig. S6*A*). Like its homologs in other vertebrates, tilapia Foxp3 contains 11 coding-exons and ten introns (Fig. S6*B*). Both tilapia and mouse Foxp3s encode two functional domains charactering the FOXP family, namely zinc finger domain and fork-head domain (Fig. S6*C*). The fork-head domain of tilapia Foxp3 shares high similarities of primary and tertiary structures with its counterparts in other vertebrates (Fig. S6, *D* and *E*). And the phylogenetic analysis confirms that tilapia Foxp3 is an orthologous molecule of vertebrates’ Foxp3s (Fig. S6*F*).

**Figure 8:**
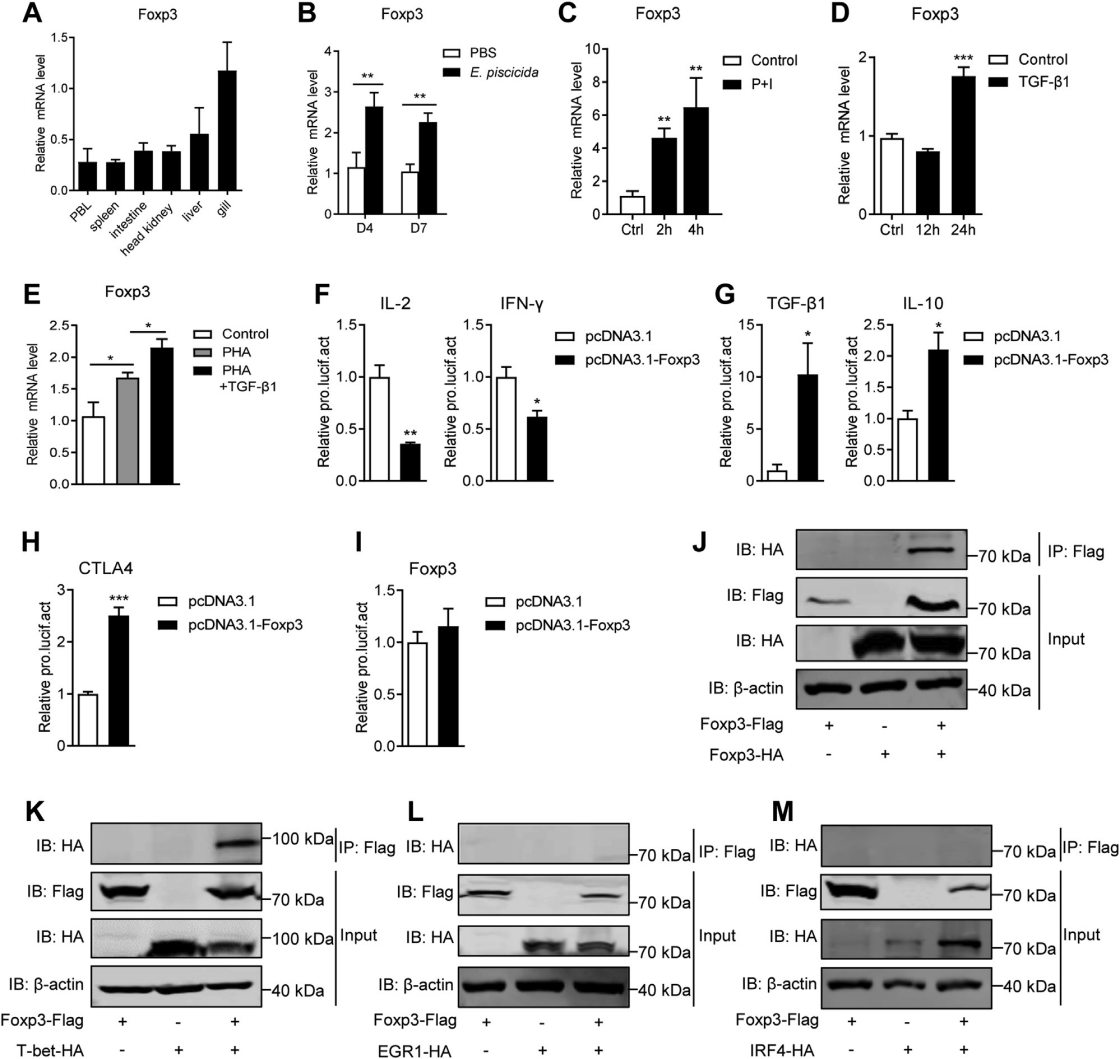
**Tilapia TGF-β1 initiates Foxp3 to transcriptionally restrain T-cell response.***A*, Foxp3 expression in the indicated tissues was analyzed by qPCR, n = 5. *B*–*D*, the relative mRNA level of Foxp3 in spleen lymphocytes isolated from *E. piscicida*–infected tilapia (*B*, n = 6), or stimulated with P + I (*C*, n = 4) or recombinant MP-TGF-β1 (*D*, n = 6) at indicated time points. *E*, spleen lymphocytes that stimulated with PHA were treated with or without recombinant MP-TGF-β1, and relative mRNA levels of Foxp3 were examined at 12 h. *F*–*I*, HEK 293T cells were cotransfected with tilapia Foxp3 and pGL3-IL-2 (IFN-γ, TGF-β1, IL-10, CTLA4, or Foxp3) promoter. The LUC activities were assessed at 48 h posttransfection, n = 4. *J*–*M*, HEK 293T cells cotransfected with Foxp3-Flag-pEGFP-C1 plasmid and indicated partner-HA-pEGFP-C1 were subjected to Western blotting directly for input control or after immunoprecipitation with anti-Flag Ab-conjugated agarose beads. The interaction of Foxp3 with Foxp3 (*J*), T-bet (*K*), EGR1 (*L*), and IRF4 (*M*) were examined. The above experiments were repeated at least two independent times. ∗*p* < 0.05, ∗∗*p* < 0.01, ∗∗∗*p* < 0.001, determined by a two-tailed Student’s *t* test. IL, interleukin; MP, mature peptide; qPCR, quantitative real-time RT-PCR; TGF-β1, transforming growth factor-β1.

Tilapia Foxp3 was constitutively expressed in the lymphocyte-associated tissues/organs (Fig. 8*A*). Consist with tilapia TGF-β1 (Fig. 2, *B* and *E*), mRNA levels of Foxp3 in spleen lymphocytes were significantly upregulated upon *in vivo E. piscicida* infection or *in vitro* P + I stimulation (Fig. 8, *B* and *C*), indicating a potential association of TGF-β1 with Foxp3. The facts that TGF-β1 treatment markedly upregulated the expression of Foxp3 in spleen lymphocytes (Fig. 8*D*), and further augmented the PHA-induced upregulation of Foxp3 (Fig. 8*E*), confirmed the inducible expression of Foxp3 by TGF-β1 in tilapia. Next, we examined whether Foxp3 can restrain T-cell response of tilapia. We suggested that the LUC activity driven by IL-2 or IFN-γ promoter was significantly reduced upon Foxp3 overexpression (Fig. 8*F*), indicating the potential inhibition of Foxp3 on the T-cell activation and proliferation (54). Meanwhile, overexpression of Foxp3 enhanced the transcriptional activities of anti-inflammatory cytokines TGF-β1 and IL-10 (Fig. 8*G*), and the inhibitory receptor CTLA4 (Fig. 8*H*), but not itself (Fig. 8*I*). Foxp3 usually suppresses the target genes *via* cooperating with other transcription factors. For example, it interacts with either Foxp1 or itself to inhibit the IL-2 transcription (55, 56). Using Co-IP assay, we revealed that tilapia Foxp3 is able to form a homodimer with itself (Fig. 8*J*), further supporting its inhibition on the IL-2 transcription. In addition, T-bet, IRF-4, and EGR-1 are key transcription factors crucial for IFN-γ, IL-4, or IL-2 expression and T-cell response (26, 57, 58). Here, we found that tilapia Foxp3 could directly interact with the IFN-γ–specific transcription factor T-bet (Fig. 8*K*), but not with IRF-4 and EGR-1 (Fig. 8, *L* and *M*). Taken together, these findings indicate that TGF-β1 may suppress T-cell responses of Nile tilapia *via* inducing Foxp3, which in turn regulates the transcription of multiple T-cell genes.

## Discussion

As a typical immunosuppressive cytokine, TGF-β1 plays pivotal roles in limiting inflammatory response and maintaining immune homeostasis (59). In addition to mediating the immune tolerance to self-antigen or nonharmful pathogens, TGF-β1 directly attenuates the activation and proliferation of a variety of immune cells induced by pathogenic pathogens (17, 21). In particular, the intensive suppression of TGF-β1 on inflammation and T-cell cytotoxicity greatly relieves the pressure and functional redundancy of the immune system after infection clearance (17, 60). The dysfunction of TGF-β1 can thus lead to spontaneous activation and abnormal proliferation of T cells, which are highly associated to autoimmune diseases (2, 34). Given that, the last 2 decades have witnessed great progresses regarding TGF-β1–controlled T-cell immunity in human and mouse. However, as an ancient cytokine, TGF-β has been proved to originate about one billion years ago (61), well before the inception of the T-cell immunity. When and how the TGF-β system rewires at the molecular and cellular levels to regulate T cells remain unknown. In this study, using Nile tilapia as a model, we investigated the suppression of TGF-β1 on the immunobiological processes of primordial T cells and revealed the regulatory mechanisms, which would provide new perspectives for the evolution of both TGF-β signaling and T-cell immunity.

TGF-β1 evolutionarily emerged in deuterostomes and is widely found in vertebrates (62). As the major member that performs immune regulatory functions of the TGF-β superfamily, TGF-β1 has been reported in mammals, birds, amphibians, and many teleost fishes (63, 64). In the present study, we identified a TGF-β1 in Nile tilapia, which shares high similarities of gene synteny and structure, functional domain, and motif, and tertiary structure with its homologs in other vertebrates, indicating it may perform similar biological function with the mammalian counterparts. In mouse, TGF-β1 can be produced by a variety of immune cells (28). Here, we suggested that TGF-β1 is broadly existed in the peripheral lymphoid tissues and mucosa-associated lymphoid tissues of tilapia, and its expression in spleen lymphocytes can be markedly induced by P + I or PHA stimulation at mRNA and protein levels, suggesting lymphocytes and T cells are pivotal cellular source of tilapia TGF-β1. Previous studies revealed that LPS or Poly I:C stimulation or *Streptococcus agalactiae* infection could induce an upregulated expression of TGF-β1 mRNA in head kidney leukocytes of common carp and grass carp (45, 65). In this study, similar results were also found in spleen leukocytes of tilapia upon *E. piscicida* infection. Therefore, it can be speculated that teleost TGF-β1 plays an important role in the immune response against pathogen infection. However, to date, the regulation of T-cell immunity by TGF-β1 has not been well addressed in teleost.

TGF-β1–deficiency mice display a neonatal lethal inflammatory disease associated with the production of multiple effector cytokines (60). Given that T cell–specific deficiency of TGF-βRII or TGF-βRI develops similar phenotypes, T cells have been regarded as the central direct target of TGF-β1–regulated immune tolerance (59, 66). Corroborating by the emerging findings, TGF-β1 is pervasive and essential for the T-cell development, homeostasis, tolerance, proliferation, differentiation, and effector function (67). Blockade of TGF-β1 signaling results in a spontaneous activation of T cells, which infiltrate in healthy tissues and cause a marked inflammatory response, representing by increased expression of FasL, perforin, granzymes, and IFN-γ (66). Consistent with this, we found that TGF-β1 suppressed the inflammatory properties of spleen lymphocytes in tilapia to maintain the immune homeostasis. Furthermore, TGF-β1 administration impaired the activation and proliferation of tilapia T cells and compromised their ability to produce the cytotoxic and proinflammatory cytokines during bacterial infection. Similar with that in mammals (68), tilapia TGF-β1 might achieve these T-cell suppressions by limiting IL-2 expression. Meanwhile, the enhanced apoptosis in the presence of TGF-β1 also contributed to the impaired expansion of tilapia T cells. Interestingly, TGF-β1 seems to perform distinct functions under different cellular contexts. For example, it may promote the survival of naive CD4^+^ T cells *in vivo* (69) and can even block FasL-mediated T-cell apoptosis in combination with IL-2 (70). In addition, TGF-β1 is able to inhibit Th1 and Th2 differentiation but promotes the development of Th17 cells in the presence of IL-6 (67). In this study, we revealed that tilapia TGF-β1 itself significantly inhibits the expression of transcription factors associated with Th1, Th2, and Th17 lineages. However, whether collaboration of TGF-β1 and IL-6 could promote the Th17 differentiation of tilapia remains unknown. Therefore, TGF-β1 exhibits profound inhibitory effects on T-cell activation, proliferation, differentiation, and function in Nile tilapia. Given that grass carp TGF-β1 can also suppress the proliferation of Zap70^+^ cells, it is reasonable to speculate that TGF-β1 already evolved to work with T cells prior to the divergence of tetrapod lineage from the teleost.

Mammalian TGF-β1 controls T-cell response *via* receptor-mediated activation of Smad transcription factors. Engagement of TGF-β1 and TGF-βR2 leads to the conformational activation of TGF-βR1 and in turn initiates the downstream Smad2/3/4, serving as a central conduit and regulating the transcriptional output of T cells (71). Both receptors and Smad proteins are crucial for maintaining the strength and sustainability of TGF-β1 signaling (50). In mouse, deficiency of Smad2 or Smad3 results in an enhanced Th1 response and develops lethal autoimmune diseases early in life (72), while nonobese diabetic mice lacking Smad4 exhibits spontaneous activation of CD4^+^ T cells, accompanying by increased serum IFN-γ and auto-antibodies, which in turn accelerated the disease progression (73). Teleost species, such as zebrafish and flounder, were also found to possess TGF-β/Smad signaling (35, 74). In this study, tilapia was found to encode evolutionarily conserved TGF-βR1, TGF-βR2, and Smad2/3/4, whose expression or phosphorylation could be induced by PHA or TGF-β1. In addition, such activation was strongly inhibited upon TGF-βR1 blockade. These observations indicated the existence of intact TGF-βR/Smad axis ensuring the transduction of TGF-β1 signaling in tilapia T cells. Actually, Smad complexes bind to DNA weakly, and high-affinity DNA binding is achieved by the association of Smad proteins with a variety of transcription factor partners (75). Here, we revealed that upon activation, tilapia Smad2/3 translocated into the nucleus, where they formed transcriptional complexes with NFAT1, T-bet, and Foxp3 for targeting gene transcription. More importantly, overexpression of tilapia Smad3 compromised the promoter activities of IL-2 and IFN-γ but enhanced the transcriptional activities of typical negative regulators such as CTLA4 and Foxp3, which accounts for the suppression of T-cell response by TGF-β1/Smad3 signaling in Nile tilapia. To our knowledge, these findings represent the first description regarding the detail mechanism by which TGF-β1 suppresses T-cell immunity in teleost species.

Driving T_reg_ cells development and maintaining their function is another strategy of TGF-β1 to suppress T-cell immunity. The accumulating evidence showing reduced Foxp3^+^ T_reg_ cells in TGF-β1 or TGF-βR2-deficiency mouse (66) highlights the important roles of TGF-β1 signaling in maintaining T_reg_ lineage. The stability and inhibitory function of T_reg_ cells rely on the transcription factor Foxp3 (76). Similar to that in TGF-β1–deficiency mouse, T cell–specific mutation or deficiency of Foxp3 results in lethal autoimmune diseases early in the life (77), whereas conditional knockout of Foxp3 in CD4^+^ T cells causes severe lymphoproliferative diseases (78). On the contrary, retroviral gene transfer of Foxp3 converts naïve T cells toward a T_reg_ cells phenotype similar to that of naturally occurring CD4^+^ T_reg_ cells (78). In tilapia, we found that T-cell activation elevated the Foxp3 transcription, and this inducible expression was more obvious once TGF-β1 existed. In addition, overexpression of the Smad3 that is downstream of TGF-β1 dramatically enhanced the promoter activity of Foxp3. More importantly, Foxp3 in turn inhibited the transcription of IL-2 and IFN-γ by forming homodimers and directly interacting with T-bet but increased the promoter activity of repressors such as TGF-β1, IL-10, and CTLA4. Therefore, our findings support a conclusion that TGF-β1 suppresses T-cell immunity in tilapia by triggering a transcriptional network composed of Smad3 and Foxp3. Considering the existence of T_reg_ subsets in zebrafish (79), we speculate that TGF-β1 may also promote the differentiation or function of Foxp3^+^ T_reg_ cells, thereby mediating the immunosuppression of tilapia T cells.

Notably, compared to the advances in mammals, our results in tilapia proposed a more detailed negative regulatory network for T-cell immunity–mediated by the TGF-β1–Smad3-Foxp3 axis. To our knowledge, Smad3 interacts with NFAT1 and T-bet to inhibit IL-2 and IFN-γ expression but to promote the CTLA4 transcription has not been reported in mammals, while TGF-β1 induces Foxp3 expression, which in turn promotes the transcription of TGF-β1 and IL-10 *via* a feedback loop and also represents the first description in vertebrates. Although whether such regulatory mechanisms also exist in mammals remains further confirmation, these results to some extent have provided new evidence for understanding the mechanism by which TGF-β1 inhibits T-cell immunity.

**Figure 9:**
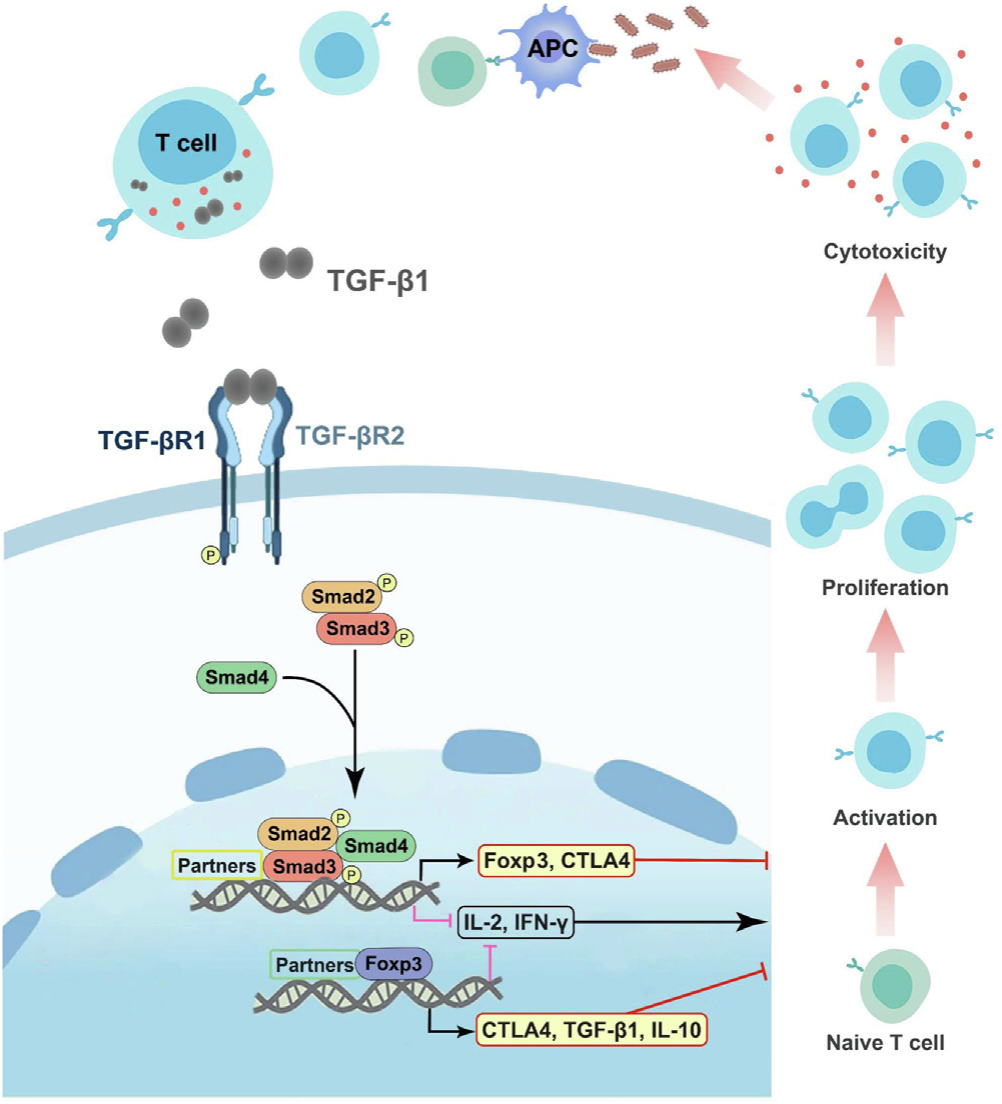
**TGF-β1 suppresses T-cell response of tilapia by initiating Smad3- and Foxp3-mediated transcriptional networks.** Upon bacterial infection, the activated T cells of tilapia produce TGF-β1, which initiates TGF-βR/Smad axis and triggers nuclear input of Smad2/3. Smad3 interacts with transcriptional partners to repress IL-2 and IFN-γ transcription but promote CTLA4 and Foxp3 transcription. Moreover, TGF-β1/Smad axis employs Foxp3 to facilitate the cascade regulation of these T-cell genes. Using this transcriptional network, TGF-β1 achieves the inhibition of T-cell activation, proliferation, and function in Nile tilapia. IL, interleukin; TGF-β1, transforming growth factor-β1.

In summary, we revealed a detail mechanism by which TGF-β1 suppresses the T-cell immune response in the teleost Nile tilapia (Fig. 9). Upon bacterial infection, tilapia T cells produce TGF-β1, which in turn initiates TGF-βR/Smad signaling pathway and results in the nuclear translocation of Smad2/3. In the nucleus, Smad3 interacts with transcriptional partners to promote or repress the transcription of T cell–related genes such as IL-2, IFN-γ, CTLA4, and Foxp3. Moreover, TGF-β1/Smad signaling further utilizes Foxp3 to achieve the cascade regulation of these T-cell genes. With the help of the transcriptional network composed of Smad3 and Foxp3, TGF-β1 inhibits the T-cell activation, proliferation, potential differentiation, and effector function, thus preventing the occurrence of excessive inflammation during antibacterial immune response. Our findings suggest that the ancient TGF-β1 system already rewired at the molecular and cellular levels to suppress T-cell immunity in some teleost species and would shed new light on the evolution of TGF-β signaling and T-cell immunity.

## Experimental procedures

### Ethics statement

All experiments involved in this study were conducted according to the Guide for the Care and Use of Laboratory Animals issued by the Ministry of Science and Technology of China and ratified by the Experimental Animal Ethics Committee of East China Normal University, with an approval number m20190108. All efforts were done to alleviate the suffering of experimental animals.

### Experimental animals

Nile tilapia larvae with 3-cm body length were purchased from the aquatic farm in Guangzhou, Guangdong Province. All fish were reared in the aerated freshwater circulating system at 28 °C and fed with commercial pellets daily until the body length reaching 8 to 10 cm. The healthy individuals were chosen randomly for the study. BALB/c mice were purchased and kept in Minhang Laboratory Animal Center of East China Normal University.

### Sequence, structure, and phylogenetic analysis

All cDNA sequences and amino acid in this study were downloaded from the National Center for Biotechnology Information (https://www.ncbi.nlm.nih.gov) and analyzed by BLAST algorithm. The protein domain prediction was performed on SMART version 4.0, and sketch maps of domain organization diagrams were prepared with DOG version 2.0 software. Multiple sequence alignments were constructed using ClustalX 1.83 according to homology analysis. The tertiary structures prediction was performed on SWISS-MODEL and displayed by PyMOL software (https://pymol.org/2/). The phylogenetic analysis was conducted using the Molecular Evolutionary Genetics Analysis X (MEGAX https://www.megasoftware.net/dload_mac_beta) software with 1000 bootstraps replications. The information of all the selected genes was shown in Table S1.

### Prokaryotic protein recombination

The fragments encoding FL and MP of tilapia TGF-β1 and extracellular region of tilapia TGF-βR1 and TGF-βR2 were respectively amplified from Nile tilapia cDNA using gene-specific primers and inserted into pMD19-T simple vector (TaKaRa). After sequencing and being digested by the restriction enzymes, the segments were cloned into pET28 vector, pET43.1a vector, and pGEX-4T-3 vector, respectively. The FL-TGF-β1 in pET28 vector, MP-TGF-β1 in pET43.1a vector, or TGF-βR1 or TGF-βR2 in pGEX-4T-3 vector was separately transformed into *Escherichia coli* Transetta DE3 complete cells (TransGen Biotech). After sequencing by quantitative real-time RT-PCR (qPCR), the positive transformants were cultured in LB medium containing proper antibiotic at 37 °C with shaking of 220 rpm until the A_600_ value reaching 0.6. Then, 1 mmol/l IPTG was added and cultured for another 4 h. The recombinant TGF-β1 proteins were purified by a Ni^2+^-chelated sepharose column or GST-resin and analyzed by 12% SDS-PAGE. The recombinant FL-TGF-β1 was gradually renaturing in urea–tris buffer at 4 °C, while the MP-TGF-β1, TGF-βR1, or TGF-βR2 protein was dialyzed in PBS twice at 4 °C. The purified FL-TGF-β1 and MP-TGF-β1 proteins were used for polyclonal antibody generation and functional analysis, respectively. All primers used were listed in Table S2.

### Bacterial infection

*Edwardsiella piscicida* from East China University of Science and Technology was cultured in Tryptic soy broth medium at 37 °C to the exponential phase. Then, the bacteria were collected, washed, and resuspended in PBS to a concentration of 1 × 10^6^ CFU. Each tilapia was intraperitoneally (*i.p.*) injected with 200 μl of bacterial suspension, while fish injected with 200 μl PBS was used as control. When detecting the cytokine production, 6 h before sacrifice, Nile tilapia was *i.p.* injected with 13 mg/kg Brefeldin A to block cytokine secreting. To investigate the immune regulation of TGF-β1, we *i.p.* injected 5 mg/kg body weight of recombinant MP-TGF-β1 protein to the fish every day during bacterial infection. The mortality of the fish was recorded daily and the survival curve was analyzed finally.

### Lymphocytes isolation

We isolated high purity of spleen lymphocytes of Nile tilapia using Percoll density gradient centrifugation according to our previous study (38). Briefly, the spleen was grinded in precooling Leibovitz's L-15 medium (Gibco) and filtered with nylon filter. The Percoll (GE Healthcare) was mixed with 10 × PBS at a 9:1 ratio and then respectively diluted to 52% Percoll and 34% Percoll with L-15 medium. Four milliliters of 52% Percoll, 34% Percoll, and cell suspension were successively added into a 15-ml centrifuge tube. The samples were centrifuged at 500*g* with the lowest acceleration and deceleration at room temperature for 35 min, and almost all the cells gathered in the liquid surface between 52% and 34% Percoll were identified as spleen lymphocytes (38). The cells were then collected, washed twice, and resuspended in L-15 medium for further experiments.

### Lymphocytes stimulation

The spleen lymphocytes cultured in Dulbecco’s modified Eagle’s medium (DMEM) containing 10% fetal bovine serum (FBS), 1% penicillin-streptomycin were stimulated with 1 μg/ml of PHA (Solarbio) with or without 5 μg/ml recombinant MP-TGF-β1 protein for indicated time. The cells were then harvested for Western blot, qPCR, or flow cytometry assay. To investigate the activation signaling, spleen lymphocytes were resuspended in dulbecco's phosphate buffered saline and rested at 28 °C for 30 min and then stimulated with 50 ng/ml phorbol 12-myristate 13-acetate plus 500 ng/ml ionomycin (P + I) or 10 μg of recombinant MP-TGF-β1 protein for indicated timepoints. The simulation was ceased by adding precooling dulbecco's phosphate buffered saline. The cells were then collected for further assays as above. 1 μg/ml of Golgiplug was added in the solution when detecting the cytokine production.

### Specific inhibitor treatment

To inhibit TGF-β1 activity, the freshly isolated spleen lymphocytes cultured in DMEM medium containing 10% FBS and 1% penicillin and streptomycin were firstly treated with 4.5 nM TGF-β1 inhibitor SB-431542 (MedChemExpress) for 1 h, before 5 μg/ml recombinant MP-TGF-β1 protein were added. The spleen lymphocytes were then collected at indicated timepoint for the Western blot assay.

### Antibody preparation

Eight-week-old mice were *i.p.* immunized with recombinant FL-TGF-β1 protein mixed with complete Freund's adjuvant (Sigma). Two weeks later, mice were *i.p.* reimmunized with same dose of recombinant FL-TGF-β1 mixed with incomplete Freund's adjuvant (Sigma). Then, we injected mice twice *via* tail vein with the same antigen dissolved in PBS with 1 week interval. Mice were sacrificed on day 7 after the last immunization, and the serum containing anti-tilapia TGF-β1 antibodies was collected.

### Death and apoptosis assay

To investigate the cell death and apoptosis, the isolated spleen lymphocytes were stained by 1:400 diluted Annexin V antibody (BioLegend) in Annexin V binding buffer (0.01 M Hepes/NaOH, pH 7.4, 0.14 M NaCl, 2.5 mM CaCl_2_) at room temperature for 15 min. And 1:400-diluted 7AAD (Life Technologies) was added into the sample, shortly before collection on the flow cytometer.

### Plasmid construction, transfection, and Co-IP assay

The FL fragments encoding Smad3, Foxp3, NFAT1, T-bet, IRF4, and EGR1 were amplified from Nile tilapia cDNA using gene-specific primers containing HA- or Flag-tag sequences. To construct the transfection plasmids, these fragments digested by restriction enzymes were ligated into pEGFP-C1. Then, 5 μg Smad3-Flag-pEGFP-C1 or Foxp3-Flag-pEGFP-C1 plasmid and 5 μg partner-HA-pEGFP-C1 plasmid as indicated in the figures were used to cotransfect the HEK 293T cells. The cells were harvested and lysed in RIPA lysis buffer containing freshly added protease inhibitors at 48 h posttransfection. The obtained supernatant was used for Co-IP assay using anti-Flag Ab-conjugated agarose beads (Sigma-Aldrich). Briefly, 10 μl anti-Flag Ab-conjugated agarose beads were added into the lysate of transfected 293T cells and incubated at 4 °C with shaking overnight. After washing with lysis buffer for four times, the beads were heated at 95 °C in SDS loading buffer for 10 min and subjected to Western blot assay. The used primers were listed in Table S2.

### Dual-luciferase reporter assay

The coding region of Nile tilapia Smad3 or Foxp3 was cloned into the pcDNA3.1 vector, while the promoter region (−2000 to 0 bp) of IL-2, IFN-γ, IL-10, TGF-β, CTLA4, or Foxp3 was ligated into pGL3 vector. The HEK 293T cells were cultured in a 24-well plate with a density of 1.5 × 10^5^/well. After 12 h, the cells were moved into FBS-free DMEM medium and cotransfected with reporter genes (pGL3-promote), transcription factor expression plasmid (pcDNA3.1-Fopx3 or pcDNA3.1-Smad3), and PRL-TK in opti-DMEM containing 1 μl Lipofectamine 2000 (Invitrogen). The medium was changed to DMEM medium containing 1% penicillin-streptomycin and 10% FBS at 6 h after transfection. At 48 h after transfection, the cells were collected and lysed with 100 μl passive lysis buffer buffer. Luc activity was analyzed using pGL3 Dual-Luciferase Reporter System (Promega) according to the manufacturer’s instructions.

### Quantitative real-time RT-PCR

TRIzol reagent (Invitrogen) was used to extract the total RNA from spleen lymphocytes or the indicated tissues of Nile tilapia according to the manufacturer's instructions. The RNA was reverse-transcribed to cDNA using Plus All-in-one 1st Strand cDNA Synthesis SuperMix (Novoprotein). Using diluted cDNA as template, qPCR was performed with 2 × NovoStart SYBR qPCR SuperMix Plus (Novoprotein) on the QuantStudio5 Q5 (Applied Biosystems). β-actin was chosen as internal control, and the relative mRNA levels of target genes were calculated by the 2^−△△CT^ method. The used primers for qPCR were listed in the Table S2.

### Western blot assay

The spleen lymphocytes were lysed in RIPA lysis buffer, and nuclear proteins were extracted using Nuclear Protein Extracting Kit (Beyotime) following the manufacturer’s protocols. The obtained protein samples were boiled in protein sample buffer (Solarbio) at 100 °C for 5 min, separated by 12% SDS-PAGE, and transferred onto nitrocellulose membranes at 100 V for 2 h. The nitrocellulose membranes were blocked with 4% nonfat powder in PBS-T (PBS with 0.05% Tween-20) at room temperature for 1 h. Then, the membranes were incubated with 1:1000 diluted primary antibody, including anti-β-actin (CST, Cat No. #3700), anti-Smad2/3 (CST, Cat No. #8685), anti-p-Smad2 Ser465/Ser467 (CST, Cat No. #18338), anti-p-Smad3 Ser423/425 (CST, Cat No. #9520), anti-p-AKT Thr308 (CST, Cat No. #13038), anti-p-ERK1/2 Thr-202/Tyr-204 (CST, Cat No. #4370), anti-p-S6 Ser240/244 (CST, Cat No. #5364), anti-Histone H3 (CST, Cat No. #9717), or anti-tilapia TGF-β1 serum at 4 °C overnight. Then, the blots were washed for three times with PBS-T before incubating with 1:30,000-diluted Fluor 800–conjugated goat anti-rabbit IgG H&L Alexa (CST) or 1:10,000-diluted Alexa Fluor 680–conjugated goat anti-mouse IgG H&L (Abcam) at room temperature for 1 h. After washing with PBS-T for three times, the blots were scanned using Odyssey CLx Image Studio.

### GST pull-down

The purified GST-tag TGF-βR1 or TGF-βR2 was incubated in GST-resin with a slight rotation at room temperature for 2 h. After three times of wash, the recombinant His-tag TGF-β1 was added and gently rotated at room temperature for another 2 h. After removing extra protein and washing for three times, the GST-resin was then suspended in SDS-loading buffer and denatured at 95 °C for 10 min. The samples were then subjected to SDS-PAGE assay.

### Flow cytometry and cell sorting

Freshly isolated spleen lymphocytes were resuspended in flow cytometry and cell sorting (FACS) buffer (PBS containing 2% FBS) for FACS analysis. The CD3^+^ T cells, CD4-1^+^CD3^+^ T cells, and IgM^+^ B cells were identified according to our previous reports (80, 81). Briefly, the lymphocytes were first stained with FITC-labeled anti-tilapia CD3ε mAb and biotin-labeled anti-tilapia IgM mAb or biotin-labeled anti-tilapia CD4-1 mAb on ice for 30 min and washed twice with FACS buffer. Then, the cells were stained with APC-conjugated streptavidin (BioLegend) on ice for 30 min, washed twice, and resuspended in FACS buffer for assay. All samples were collected using a BD Biosciences CantoII flow cytometer, and results were analyzed by FlowJo software (https://www.bdbiosciences.com/zh-cn/products/software/flowjo-v10-software). For cell sorting, spleen lymphocytes were stained with CD3 mAb as above, and CD3^+^ T cells were sorted using a BD FACSAria II flow cytometer.

### BrdU incorporation

The bacteria-infected or uninfected tilapia individuals were *i.p.* injected with 0.75 mg BrdU (BD Pharmingen) in 200 μl PBS 1 day before the spleen lymphocytes were isolated for assays on day 5 postinfection. The cells were fixed with BD Cytofix/Cytoperm buffer on ice for 30 min and washed twice using BD Perm/Wash Buffer. Then, BD Cytoperm Plus Buffer was added and placed on ice for 10 min to permeabilize the cells. After two times of washing, the cells were subsequently refixed with BD Cytofix/Cytoperm Buffer at room temperature for another 5 min and washed twice again. After treating with 300 μg/ml DNase at 37 °C for 1 h, the cells were intracellularly stained with 1:100-diluted FITC-conjugated anti-BrdU antibody (BD) at room temperature for 20 min. After two times of washing, the samples were analyzed by flow cytometry.

### *In vitro* proliferation assay

Spleen lymphocytes were labeled with 10 μM CFSE (Invitrogen) at room temperature according to the manufacturer’s protocol. The labeled cells were cultured in DMEM containing 10 μg/ml PHA and 5 μg/ml recombinant tilapia IL-2 (81), with or without 10 μg/ml recombinant TGF-β1 at 28 °C for 72 h. Cells were then stained with anti-tilapia CD3ε mAb as described above, and 7-AAD was added to identify live/dead cells. The proliferation of CD3^+^ T cells was then examined by flow cytometry.

### Binding of TGF-β1 to T cells

To investigate the potential target cells of TGF-β1, spleen lymphocytes were incubated with 1 μg recombinant His-tag TGF-β1 on ice for 30 min. After two times of wash with FACS buffer, 1:1000-diluted mouse anti-His antibody (BBI life sciences) and 1:2000-diluted Alexa Fluor 405–conjugated goat anti-mouse IgG H&L (Abcam) were successively added and incubated on ice for another 30 min. Finally, the samples were stained with anti-tilapia CD4-1 and anti-tilapia CD3ε mAbs as described above. The stained cells were resuspended with FACS buffer and analyzed by flow cytometry.

### Statistical analysis

Prism version 8.0 software (GraphPad Software Inc) was used for statistical analysis. The statistical significance of results was analyzed using two-tailed, unpaired, Student’s *t* test. Animal survival data were analyzed by log-rank analysis. The significance was indicated by ∗*p* < 0.05, ∗∗*p* < 0.01, ∗∗∗*p* < 0.001.

## Data availability

The data that support the findings of this study are available in the article and supplementary material of this article.

## Supporting information

This article contains supporting information.

## Conflict of interest

The authors declare that they have no conflicts of interest with the contents of this article.
