# Supplementary material for: TGF-β1 suppresses the T-cell response in teleost fish by initiating Smad3- and Foxp3-mediated transcriptional networks
Source: J Biol Chem. 2022 Dec 26;299(2):102843. doi: 10.1016/j.jbc.2022.102843 (PMC9860442; doi:10.1016/j.jbc.2022.102843)
Supplement: Supporting Figure S1 [file mmc1.pdf]

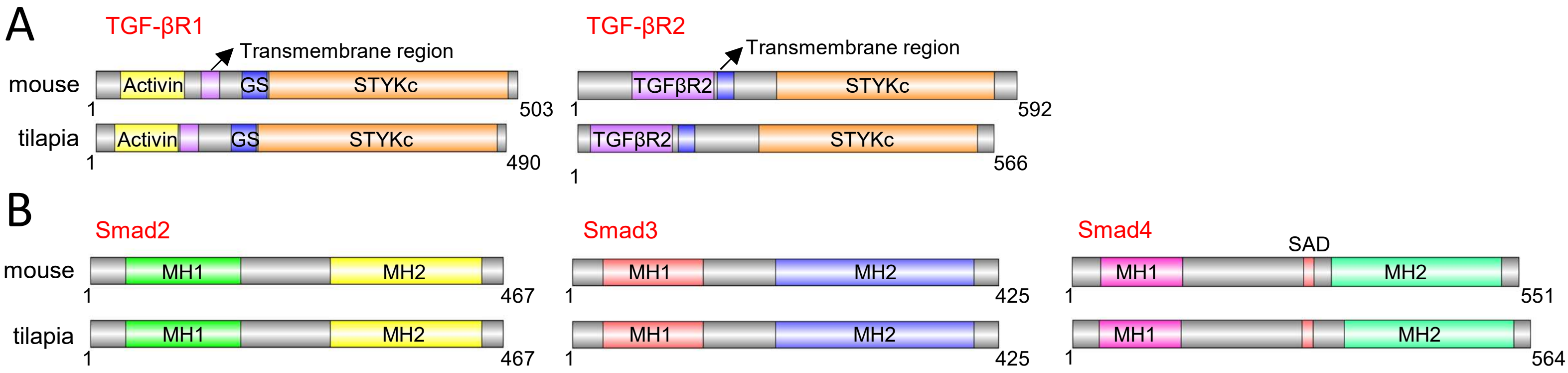

Figure S1. Domain organization of TGF-β1R/Smad components. Comparison of the domain organization of TGF-β1R1, TGF-β1R2, Smad2, Smad3 and Smad4 from Nile tilapia and mouse. The accession numbers of selected sequences are listed in Table S1.
