# Supplementary material for: TGF-β1 suppresses the T-cell response in teleost fish by initiating Smad3- and Foxp3-mediated transcriptional networks
Source: J Biol Chem. 2022 Dec 26;299(2):102843. doi: 10.1016/j.jbc.2022.102843 (PMC9860442; doi:10.1016/j.jbc.2022.102843)
Supplement: Supporting Figure S2 [file mmc2.pdf]

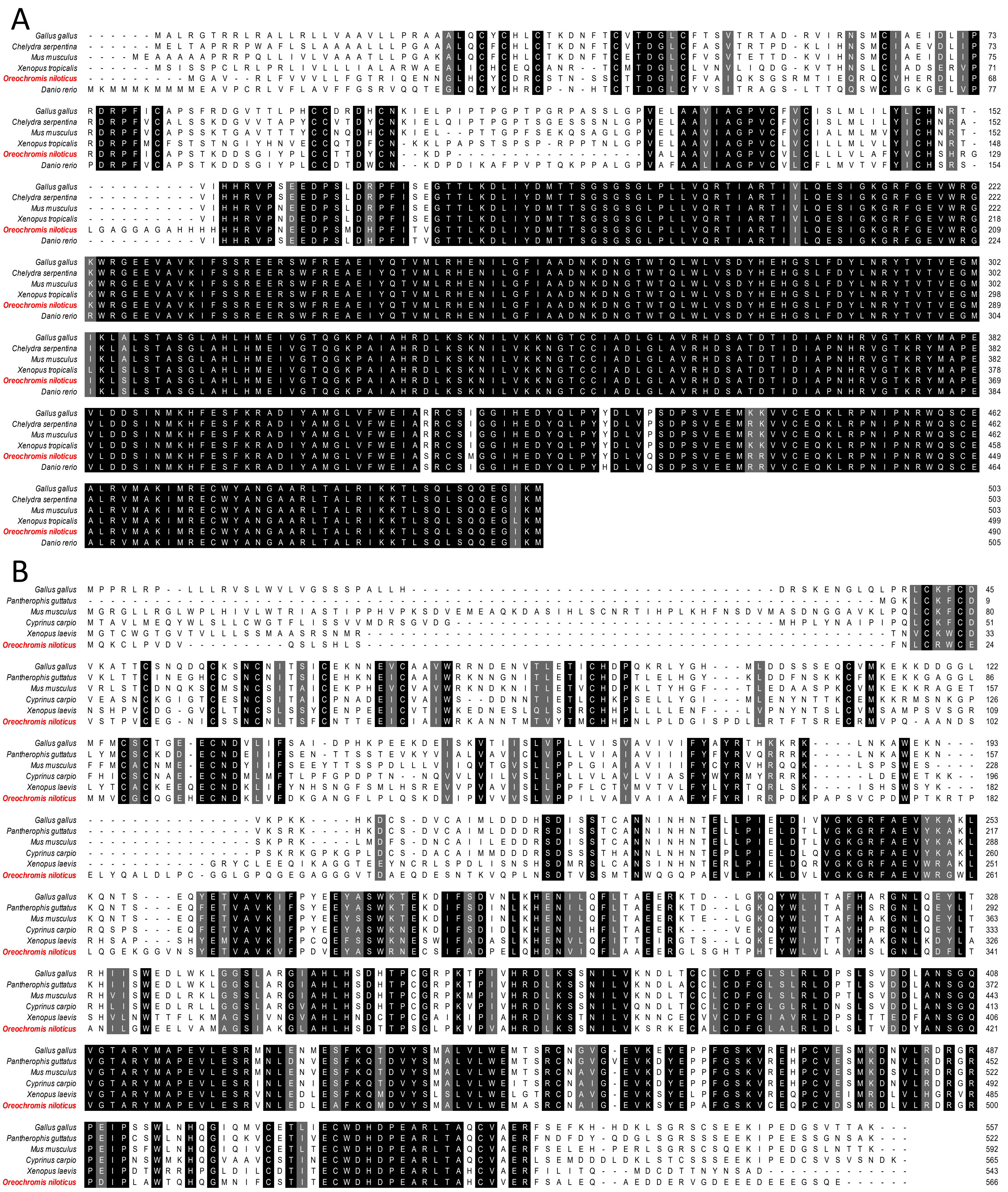

Figure S2. Multisequence alignment analysis of tilapia TGF-βRs. Multisequence alignment analysis of TGF-βR1 (**A**) and TGF-βR2 (**B**) from Nile tilapia with homologs from other animals. Amino acid residues with 100% identity are in black, and similar amino acids are in grey. The accession numbers of selected sequences are listed in Table S1.
