# Supplementary material for: TGF-β1 suppresses the T-cell response in teleost fish by initiating Smad3- and Foxp3-mediated transcriptional networks
Source: J Biol Chem. 2022 Dec 26;299(2):102843. doi: 10.1016/j.jbc.2022.102843 (PMC9860442; doi:10.1016/j.jbc.2022.102843)
Supplement: Supporting Figure S3 [file mmc3.pdf]

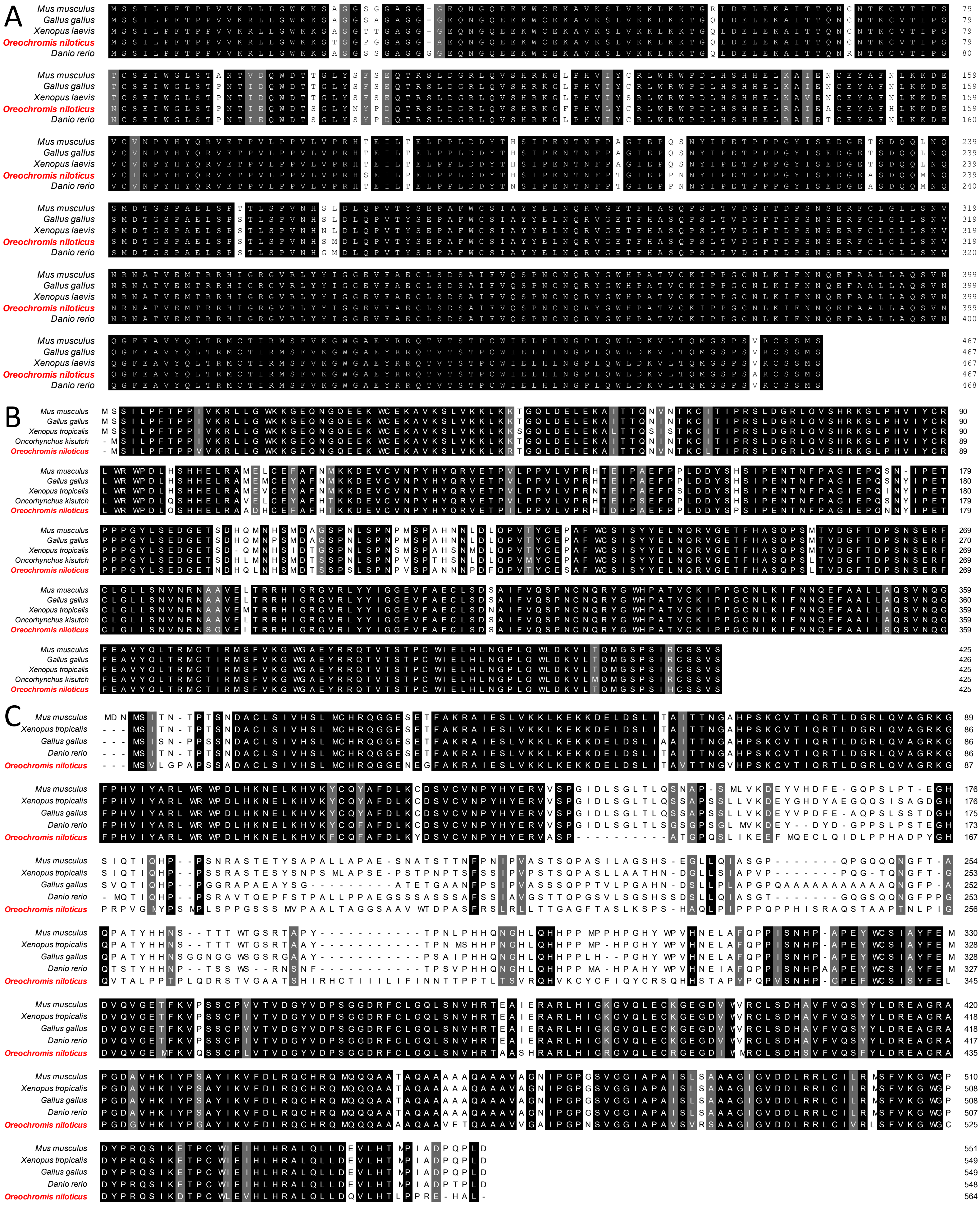

Figure S3. Multisequence alignment analysis of tilapia Smads. Multiple sequence alignment analysis of Smad2 (**A**), Smad3 (**B**) and Smad4 (**C**) from Nile tilapia with homologs from indicated species. Amino acid residues with 100% identity are in black, and similar amino acids are in grey. The accession numbers of selected sequences are listed in Table S1.
