# Supplementary material for: TGF-β1 suppresses the T-cell response in teleost fish by initiating Smad3- and Foxp3-mediated transcriptional networks
Source: J Biol Chem. 2022 Dec 26;299(2):102843. doi: 10.1016/j.jbc.2022.102843 (PMC9860442; doi:10.1016/j.jbc.2022.102843)
Supplement: Supporting Figure S4 [file mmc4.pdf]

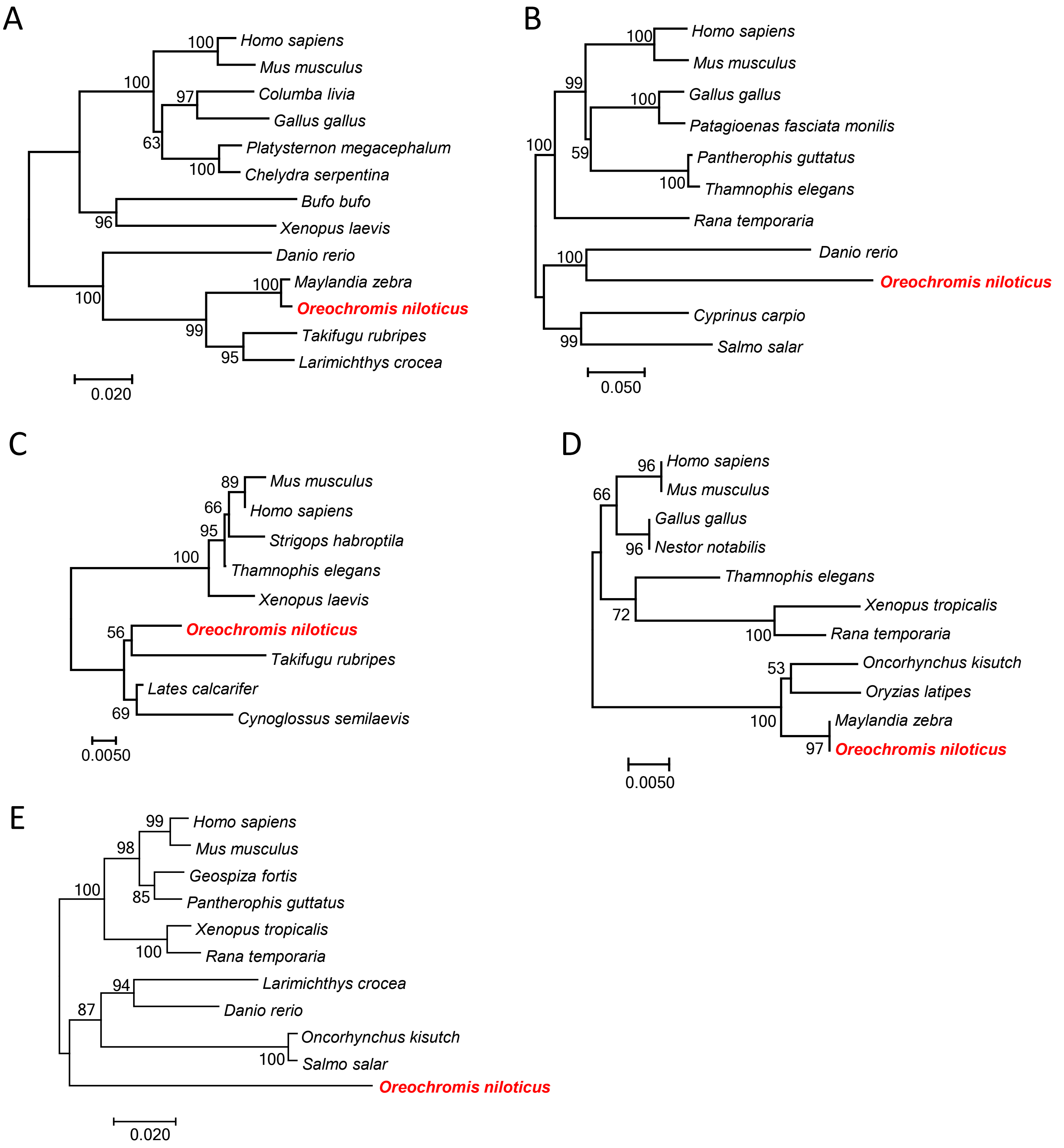

Figure S4. Phylogenetic analysis of TGF-β1R/Smad signaling components in tilapia. Phylogenetic trees of TGF-βR1 (**A**), TGF-βR2 (**B**), Smad2 (**C**), Smad3 (**D**) and Smad4 (**E**) were constructed by the neighbor-joining algorithm in MEGAX software based on multiple sequence alignment by ClustalW. Bootstrap values of 1000 replicates (%) are indicated for the branches. The accession numbers of selected sequences are listed in Table S1.
