# Supplementary material for: TGF-β1 suppresses the T-cell response in teleost fish by initiating Smad3- and Foxp3-mediated transcriptional networks
Source: J Biol Chem. 2022 Dec 26;299(2):102843. doi: 10.1016/j.jbc.2022.102843 (PMC9860442; doi:10.1016/j.jbc.2022.102843)
Supplement: Supporting Figure S5 [file mmc5.pdf]

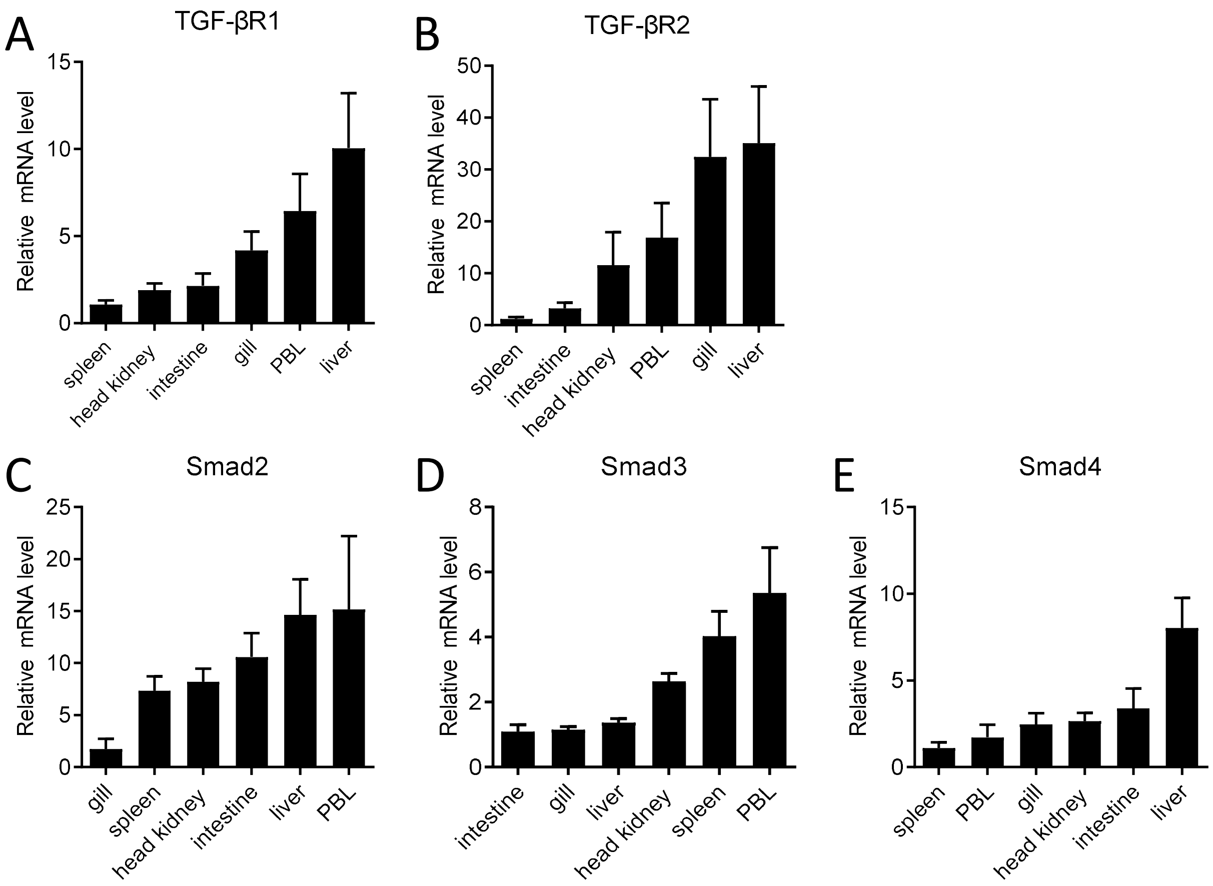

Figure S5. Tissue distribution analysis of TGF-β1R/Smad signaling components in tilapia. The relative mRNA expression levels of TGF-βR1 (**A**), TGF-βR2 (**B**), Smad2 (**C**), Smad3 (**D**), and Smad4 (**E**) in the indicated tissues were analyzed by qPCR, n=5. The experiments were repeated at least two independent times.
