# Supplementary material for: TGF-β1 suppresses the T-cell response in teleost fish by initiating Smad3- and Foxp3-mediated transcriptional networks
Source: J Biol Chem. 2022 Dec 26;299(2):102843. doi: 10.1016/j.jbc.2022.102843 (PMC9860442; doi:10.1016/j.jbc.2022.102843)
Supplement: Supporting Figure S6 [file mmc6.pdf]

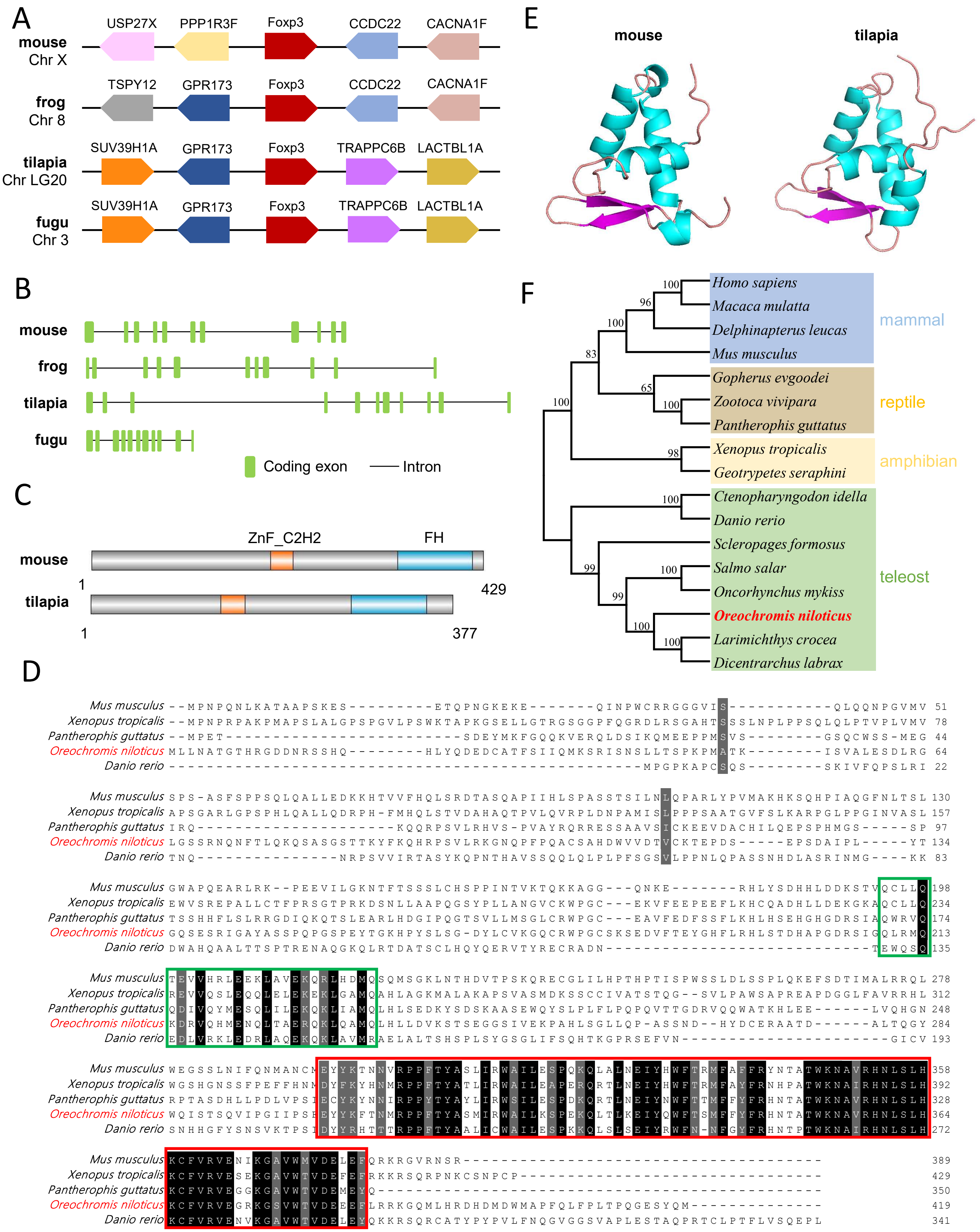

A
mouse
Chr X
USP27X
PPP1R3F
Foxp3
CCDC22
CACNA1F
frog
Chr 8
TSPY12
GPR173
Foxp3
CCDC22
CACNA1F
tilapia
Chr LG20
SUV39H1A
GPR173
Foxp3
TRAPPC6B
LACTBL1A
fugu
Chr 3
SUV39H1A
GPR173
Foxp3
TRAPPC6B
LACTBL1A
B
mouse
frog
tilapia
fugu
Coding exon
Intron
C
ZnF_C2H2
FH
mouse
1
429
tilapia
1
377
D
Mus musculus
Xenopus tropicalis
Pantherophis guttatus
Oreochromis niloticus
Danio rerio
E
mouse
tilapia
F
Homo sapiens
Macaca mulatta
Delphinapterus leucas
Mus musculus
mammal
Gopherus evgoodei
Zootoca vivipara
Pantherophis guttatus
reptile
Xenopus tropicalis
Geotrypetes seraphini
amphibian
Ctenopharyngodon idella
Danio rerio
Scleropages formosus
Salmo salar
Oncorhynchus mykiss
Oreochromis niloticus
Larimichthys crocea
Dicentrarchus labrax
teleost

Figure S6. The sequential properties of Foxp3 in Nile tilapia. A-C, Comparison of collinearity and chromosomal location (**A**), genomic structure (**B**), and domain organization (**C**) of Foxp3 from Nile tilapia and indicated vertebrates were shown. **D**, Multisequence alignment analysis of Foxp3 from Nile tilapia and other animals. Amino acid residues with 100% identity are in black, and similar amino acids are in grey. Green box: ZnF C2H2 domain; Red box: FH domain. **E**, The tertiary structures of FH domains in Foxp3 from Nile tilapia and mouse were predicted by SWISS-MODEL software. **F**, Phylogenetic tree of Foxp3 from the indicated species were constructed by the neighbor-joining algorithm in MEGAX software based on multiple sequence alignment by ClustalW. Bootstrap values of 1000 replicates (%) are indicated for the branches. The accession numbers of selected sequences are listed in Table S1.
