# Supplementary material for: TGF-β1 suppresses the T-cell response in teleost fish by initiating Smad3- and Foxp3-mediated transcriptional networks
Source: J Biol Chem. 2022 Dec 26;299(2):102843. doi: 10.1016/j.jbc.2022.102843 (PMC9860442; doi:10.1016/j.jbc.2022.102843)
Supplement: Supporting Table S1 [file mmc7.pdf]

Table S1. Information of genes used for sequence, phylogeny and structure analysis in present study

Source	Accession No.	Gene name	Species	Application
GenBank	AKA60246.1	TGF-β1	*Oreochromis niloticus*	DP,DOA,3D,PA,MSA
GenBank	AAA36738.1	TGF-β1	*Homo sapiens*	PA
GenBank	AAA37674.1	TGF-β1	*Mus musculus*	DOA,3D,PA,MSA
GenBank	XP_003222953.1	TGF-β1	*Anolis carolinensis*	PA
GenBank	XP_007421553.1	TGF-β1	*Python bivittatus*	PA
GenBank	AFD30526.1	TGF-β1	*Gallus gallus*	MSA,PA
GenBank	XP_005987924.1	TGF-β1	*Latimeria chalumnae*	PA
GenBank	XP_035535886.1	TGF-β1	*Morone saxatilis*	PA
GenBank	AEE90023.1	TGF-β1	*Branchiostoma japonicum*	PA
GenBank	XP_002939433.1	TGF-β1	*Xenopus tropicalis*	MSA,PA
GenBank	AAO60240.1	TGF-β1	*Danio rerio*	MSA,PA
GenBank	ACV96791.1	TGF-β1	*Epinephelus coioides*	PA
GenBank	ABX38812.1	TGF-β1	*Crassostrea ariakensis*	PA
GenBank	CDS32461.1	TGF-β1	*Hymenolepis microstoma*	PA
GenBank	XP_030075415.1	TGF-β1	*Microcaecilia unicolor*	PA
GenBank	ATW73239.1	TGF-β1	*Quasipaa boulengeri*	PA
GenBank	XP_011414113.1	TGF-β1	*Crassostrea gigas*	PA
GenBank	CAA07707.1	TGF-β1	*Oncorhynchus mykiss*	PA
GenBank	AWO98615.1	TGF-β1	*Scophthalmus maximus*	PA
GenBank	XP_004075270.1	TGF-β1	*Oryzias latipes*	PA
GenBank	AKA60247.1	TGF-βR1	*Oreochromis niloticus*	DP,DOA,3D,PA,MSA
GenBank	EDL02353.1	TGF-βR1	*Mus musculus*	DOA,3D,PA,MSA
GenBank	ACZ58375.1	TGF-βR1	*Homo sapiens*	PA
GenBank	KAG6925168.1	TGF-βR1	*Chelydra serpentina*	PA,MSA
GenBank	XP_040287676.1	TGF-βR1	*Bufo bufo*	PA
GenBank	XP_004572123.2	TGF-βR1	*Maylandia zebra*	PA
GenBank	TFK01883.1	TGF-βR1	*Platysternon megacephalum*	PA
GenBank	XP_029687801.1	TGF-βR1	*Takifugu rubripes*	PA
GenBank	XP_027129977.1	TGF-βR1	*Larimichthys crocea*	PA
GenBank	XP_041422104.1	TGF-βR1	*Xenopus laevis*	PA,MSA
GenBank	NP_989577.1	TGF-βR1	*Gallus gallus*	PA,MSA
GenBank	PKK24728.1	TGF-βR1	*Columba livia*	PA
GenBank	NP_001108531.1	TGF-βR1	*Danio rerio*	PA,MSA
GenBank	XP_013129468.1	TGF-βR2	*Oreochromis niloticus*	DP,DOA,3D,PA,MSA
GenBank	NP_033397.3	TGF-βR2	*Mus musculus*	DOA,3D,PA,MSA
GenBank	NP_001020018.1	TGF-βR2	*Homo sapiens*	PA
GenBank	XP_042597418.1	TGF-βR2	*Cyprinus carpio*	PA,MSA
GenBank	XP_034259994.1	TGF-βR2	*Pantherophis guttatus*	PA,MSA
GenBank	XP_040209024.1	TGF-βR2	*Rana temporaria*	PA
GenBank	XP_032093289.1	TGF-βR2	*Thamnophis elegans*	PA

GenBank	OPJ82048.1	TGF-βR2	*Patagioenas fasciata monilis*	PA
GenBank	NP_001081330.1	TGF-βR2	*Xenopus laevis*	PA,MSA
GenBank	NP_990759.1	TGF-βR2	*Gallus gallus*	PA,MSA
GenBank	NP_001133728.1	TGF-βR2	*Salmo salar*	PA
GenBank	XP_009292206.2	TGF-βR2	*Danio rerio*	PA,MSA
GenBank	XP_025752701.1	Smad2	*Oreochromis niloticus*	DP,DOA,3D,PA,MSA
GenBank	NP_001239410.1	Smad2	*Mus musculus*	DOA,3D,PA,MSA
GenBank	NP_001003652.1	Smad2	*Homo sapiens*	PA
GenBank	XP_030367053.1	Smad2	*Strigops habroptila*	PA
GenBank	XP_030593153.1	Smad2	*Archocentrus centrarchus*	PA
GenBank	XP_018524763.1	Smad2	*Rana temporaria*	PA
GenBank	XP_032069194.1	Smad2	*Thamnophis elegans*	PA
GenBank	XP_029685949.1	Smad2	*Takifugu rubripes*	PA
GenBank	XP_041430814.1	Smad2	*Xenopus laevis*	PA,MSA
GenBank	NP_989892.1	Smad2	*Gallus gallus*	PA,MSA
GenBank	NP_571441.3	Smad2	*Danio rerio*	PA,MSA
GenBank	XP_008333936.1	Smad2	*Cynoglossus semilaevis*	PA
GenBank	XP_003456800.1	Smad3	*Oreochromis niloticus*	DP,DOA,3D,PA,MSA
GenBank	NP_058049.3	Smad3	*Mus musculus*	DOA,3D,PA,MSA
GenBank	NP_005893.1	Smad3	*Homo sapiens*	PA
GenBank	NP_037227.1	Smad3	*Rattus norvegicus*	PA
GenBank	NP_999302.1	Smad3	*Sus scrofa*	PA
GenBank	NP_001192734.1	Smad3	*Bos taurus*	PA
GenBank	XP_032088757.1	Smad3	*Thamnophis elegans*	PA
GenBank	XP_010018383.1	Smad3	*Nestor notabilis*	PA
GenBank	NP_989806.1	Smad3	*Gallus gallus*	PA,MSA
GenBank	XP_031668603.1	Smad3	*Oncorhynchus kisutch*	PA,MSA
GenBank	XP_040198182.1	Smad3	*Rana temporaria*	PA
GenBank	XP_030019178.1	Smad3	*Sphaeramia orbicularis*	PA
GenBank	XP_029933816.1	Smad3	*Myripristis murdjan*	PA
GenBank	XP_029353766.1	Smad3	*Xenopus tropicalis*	PA,MSA
GenBank	XP_023808862.1	Smad3	*Oryzias latipes*	PA
GenBank	XP_004557989.1	Smad3	*Maylandia zebra*	PA
GenBank	XP_025766932.1	Smad4	*Oreochromis niloticus*	DP,DOA,3D,PA,MSA
GenBank	NP_032566.2	Smad4	*Mus musculus*	DOA,3D,PA,MSA
GenBank	NP_005350.1	Smad4	*Homo sapiens*	PA
GenBank	XP_010736488.1	Smad4	*Larimichthys crocea*	PA
GenBank	XP_014166925.1	Smad4	*Geospiza fortis*	PA
GenBank	XP_034257445.1	Smad4	*Pantherophis guttatus*	PA
GenBank	ACA58502.1	Smad4	*Danio rerio*	PA,MSA
GenBank	XP_040192975.1	Smad4	*Rana temporaria*	PA
GenBank	XP_040511206.1	Smad4	*Gallus gallus*	MSA
GenBank	XP_031673068.1	Smad4	*Oncorhynchus kisutch*	PA

GenBank	XP_014027972.1	Smad4	*Salmo salar*	PA
GenBank	XP_002934485.1	Smad4	*Xenopus tropicalis*	PA,MSA
GenBank	AGT50479.1	Foxp3	*Oreochromis niloticus*	DP,DOA,3D,PA,MSA
GenBank	NP_001186277.1	Foxp3	*Mus musculus*	DOA,3D,PA,MSA
GenBank	ABQ15210.1	Foxp3	*Homo sapiens*	PA
GenBank	XP_022419431.1	Foxp3	*Delphinapterus leucas*	PA
GenBank	NP_001028090.1	Foxp3	*Macaca mulatta*	PA
GenBank	XP_034996519.1	Foxp3	*Zootoca vivipara*	PA
GenBank	XP_030404110.1	Foxp3	*Gopherus evgoodei*	PA
GenBank	XP_034290153.1	Foxp3	*Pantherophis guttatus*	PA,MSA
GenBank	XP_031748044.1	Foxp3	*Xenopus tropicalis*	PA,MSA
GenBank	XP_033817457.1	Foxp3	*Geotrypetes seraphini*	PA
GenBank	ADO87040.1	Foxp3	*Salmo salar*	PA
GenBank	XP_018608414.1	Foxp3	*Scleropages formosus*	PA
GenBank	ADL27444.1	Foxp3	*Larimichthys crocea*	PA
GenBank	CAT65095.1	Foxp3	*Oncorhynchus mykiss*	PA
GenBank	AFH58707.1	Foxp3	*Ctenopharyngodon idella*	PA
GenBank	AIK66529.1	Foxp3	*Dicentrarchus labrax*	PA
GenBank	ACQ44666.1	Foxp3	*Danio rerio*	PA,MSA

Note:

MSA: multiple sequence alignment; PA: phylogenetic analysis; 3D: 3D structure analysis; DOA: domain organization analysis; DP: domain prediction.
