# Supplementary material for: TGF-β1 suppresses the T-cell response in teleost fish by initiating Smad3- and Foxp3-mediated transcriptional networks
Source: J Biol Chem. 2022 Dec 26;299(2):102843. doi: 10.1016/j.jbc.2022.102843 (PMC9860442; doi:10.1016/j.jbc.2022.102843)
Supplement: Supporting Table S2 [file mmc8.pdf]

Table S2. Information and sequence of the primers used in present study

Accession No.	Primer name	Forward (5'-3')	Reverse (5'-3')
KJ126772.1	RT-β-actin	CGGAATCCACGAAACCACCTA	CCAGACGGAGTATTTACGCTCA
KP754231.1	Re-FL-TGF-β1	CGGAATTCCGCAGTGTACATAATCTGTCTA	CCCTCGAGGTTAATTAGCTACACTTGCA
KP754231.1	Re-ML-TGF-β1	CGGAATTCCGCAGTGTACATAATCTGTCTA	CCCTCGAGGTTAATTAGCTACACTTGCA
XM_003456752.5	Re-Smad3-Flag	CCGCTCGAGCTATGTCAATTTTGCCTTTCACTCC	GGGGTACCTTACTTATCGTCGTCATCCTTGTAATCAGAGACGCTGGAGCAGCGG
KC153968.1	Re-Foxp3-Flag	CCGCTCGAGCTATGCCAGAGACTTCTGATGAGTAC	CCGGAATTCTTACTTATCGTCGTCATCCTTGTAATCGGGGTTTCCAGGAGAGTAA
KC153968.1	Re-Foxp3-HA	CCGCTCGAGCTATGCCAGAGACTTCTGATGAGTAC	CGGAATTCTTAAGCGTAGTCTGGGACGTCGTATGGGTAGGGGTTTCCAGGAGAG
XM_003448658.4	Re-T-bet-HA	CCGCTCGAGCTATGGGCGGCATAGGTGG	CGAATTCTTAAGCGTAGTCTGGGACGTCGTATGGGTAGTGGGTGTAATAACCATA
XM_013276256.3	Re-NFAT1-HA	CCGCTCGAGCTATGACCTCCTTTTACGACGAAA	CGGAATTCTTAAGCGTAGTCTGGGACGTCGTATGGGTATGTCTGACCTCTGGCCT
XM_003455175	Re-EGR1-HA	CCGCTCGAGCTCACCAGCAGAGGATGGCTG	CGGAATTCTTAAGCGTAGTCTGGGACGTCGTATGGGTAGCAGATCTCGATTGTCC
XM_003437882.5	Re-IRF4-HA	CCGCTCGAGCTATGAACCTGGATGAAGACAGTG	CGGAATTCTTAAGCGTAGTCTGGGACGTCGTATGGGTACTCCTGAATATGCTGC
KP754231.1	RT- TGF-β1	GACTATGAGCAGGAGGGGAGAC	CAGCAGTTGTGTGATTGGGTG
AY428948.1	RT-TNF-α	CGTCGTCGTGGCTCTTTGTT	TGGGGCTCTGTTTTGTCGC
XM_005448195.3	RT-IL-6	CTTCTCTCTATCCCCGAAACACA	TACATCAGCCATTAGCCCG
XM_005457887.3	RT-IL-1β	TGACAACTCTCAGAAAGATCT	TGGTGACTCTCCTGGTCTGA
XM_003439678.4	RT-Granzyme B	GAGCATTTTGTGGTGACTGCTGC	CACGGTCTGCGAGAGGAATAGGT
XM_025896916.1	RT-Smad2	AGAGGAGAAATGGTGCGAGA	AGGCCTGATGTATCCCACTG
XM_003456752.5	RT-Smad3	CAGTAAACCAGGGCTTCGAG	GCCCATTTGTGTAAGCACCT
XM_025911147	RT-Smad3	GGTGCAATTCCCGGTCCGAA	CTTGATGCTCTGCCGTGGGT
XM_003457515.5	RT- TGF-βR1	TGGTCCAGTCAGATCCATCA	TGATTCGCAGAGCAGTGAGT
XM_013274014.3	RT- TGF-βR2	GTCCTTGAGTCCAGGGTGAA	ACAAACCTTGGAGCCAAATG
NM_001287402.1	RT-IFN-γ	GGGTGGTGTTTTGGAGTCGT	GTAGCGAGCCTGAGTTGTTGGTG
XM_019360337.2	RT-CD122	AGCCAGATGAGAAGATGATGTTGAA	TGAGTGAGGAAATAGGAGGAGGG
XM_003448658.4	RT-T-bet	ACCTCGGTCACCCAACTAAATC	CACCCACACCTCCCTCAAAT
XM_003448073.5	RT-GATA3	AACAGCATCCTGGCTCACAT	GCTCGGACAGAGTTTCCATAGT
XM_003440430.5	RT-RORα	TGTAAGGGCTTTTTCAGGAGGA	GTTGCGGCTTGTGCGGT
KC153968.1	RT-Foxp3	CAGTCGCTTACAGGCAACG	CAAGGAGAGGAAATGATGGCT
ENSONIT00000028	RT-IL-2	ATGTCGAGACCCAGGGAAAC	CAGGCCACAGGTGACAGTTA

Note:

RT: qPCR; Re: protein recombination.
